# Identification and validation of eight estrogen-related genes for predicting prognosis of papillary thyroid cancer

**DOI:** 10.18632/aging.204582

**Published:** 2023-03-13

**Authors:** Yu Zeng, Weike Ma, Lijuan Li, Gaojian Zhuang, Guoqing Luo, Hong Zhou, Weijing Hao, Yu Liu, Fengli Guo, Mengran Tian, Xianhui Ruan, Ming Gao, Xiangqian Zheng

**Affiliations:** 1 Department of Thyroid and Neck Tumor, Tianjin Medical University Cancer Institute and Hospital, National Clinical Research Center for Cancer, Key Laboratory of Cancer Prevention and Therapy, Tianjin’s Clinical Research Center for Cancer, Tianjin 300060, China; 2 Department of Cancer Prevention Center, Tianjin Medical University Cancer Institute and Hospital, National Clinical Research Center for Cancer, Key Laboratory of Cancer Prevention and Therapy, Tianjin’s Clinical Research Center for Cancer, Tianjin 300060, China; 3 The Sixth Affiliated Hospital of Guangzhou Medical University, Qingyuan People’s Hospital, Qingyuan 511500, China; 4 Department of Thyroid and Breast Surgery, Tianjin Union Medical Center, Tianjin 300121, China; 5 Tianjin Key Laboratory of General Surgery in Construction, Tianjin Union Medical Center, Tianjin 300121, China; 6 School of Medicine, Nankai University, Tianjin 300071, China

**Keywords:** papillary thyroid cancer, estrogen-related genes, NMU, prognosis, biomarkers

## Abstract

Papillary thyroid cancer (PTC) is one of the most common malignant tumors in female, and estrogen can affect its progression. However, the targets and mechanisms of estrogen action in PTC remain unclear. Therefore, this study focuses on the relationship between estrogen-related genes (ERGs) expression and prognosis in PTC, particularly neuropeptide U (NMU), and its important role in tumor progression. Based on The Cancer Genome Atlas (TCGA) and Gene Expression Omnibus (GEO) databases, differentially expressed genes (DEGs) predominantly enriched in ERGs were identified between PTC and normal tissue. Then, we identified ERGs that contributed most to PTC prognosis, including Transducer of ERBB2 1 (TOB1), trefoil factor 1 (TFF1), phospholipase A and acyltransferase 3 (PLAAT3), NMU, kinesin family member 20A (KIF20A), DNA topoisomerase II alpha (TOP2A), tetraspanin 13 (TSPAN13), and carboxypeptidase E (CPE). In addition, we confirmed that NMU was highly expressed in PTC and explored the effect of NMU on PTC cells proliferation *in vitro* and *in vivo*. The results showed that the proliferative capacity of PTC cells was significantly reduced with NMU knockdown. Moreover, the phosphorylation levels of the Kirsten rat sarcoma virus (KRAS) signaling pathway were significantly lower with NMU knockdown. These results suggest that ERGs, especially NMU, may be novel prognostic indicators in PTC.

## INTRODUCTION

TC is the most common malignancy of the endocrine system, and its incidence continues to rise worldwide [1, 2]. Although the overall prognosis for TC is good, 5–10% of patients may die due to distant metastases [3, 4]. PTC is one of the more prevalent pathological types, accounting for 90% of all TC cases, and is often confined and asymptomatic [5]. The cause of PTC is unknown, but possible causes include benign thyroid disease, radiation, family history, iodine intake, and abnormal estrogen levels. To further improve the OS, clinicians and scientists are working to identify effective therapeutic PTC targets and uncover the main mechanisms of action in PTC development.

In addition to acting on breast tissue and the female reproductive system [6–8], estrogens can also act on thyroid tissue and are involved in the development of many thyroid disorders [9, 10]. It has been found that the direct action of estrogen on thyroid tissue can lead to the development of goiter, nodules and cancer in women [11], but the specific mechanisms of action are unclear. In addition, the incidence of PTC differed significantly between females of childbearing age and adolescent females, and was three times more common in females than in males, suggesting that the incidence of PTC may be closely related to female estrogen levels [12, 13]. Numerous studies have found that the abnormal expression and function of ER are key factors in the development and progression of hormone-related cancers and influences the efficacy of anticancer therapy [14]. In PTC, estrogen is able to activate the tyrosine kinase signaling pathways on the cell membrane, leading to poor prognosis in PTC patients [9]. The above studies suggest that PTC occurrence is closely related to estrogen levels and ER expression.

NMU, an estrogen-related gene, is originally isolated from the pig spinal cord [15], and involved in the gut-brain axis, energy homeostasis, and immune response [16]. NMU mainly exerts physiological and pathophysiological effects by binding to neuromedin U receptors 1/2 [17], including smooth muscle contraction, feeding behavior, energy homeostasis, stress response, circadian rhythmicity, inflammation development, and tumorigenesis [18–20]. There is growing evidence that aberrant NMU expression can promote cancer development and progression [21–23].

In this study, we identified DEGs for PTC and normal tissues from TCGA and GEO databases, and found that they were predominantly enriched in estrogen-responsive related pathways. We constructed a PTC risk model and used it to identify eight ERGs with relatively large impacts on PTC prognosis. Then, we selected NMU as the primary target of this study based on the differential expression and the impact on the PFI. We explored the function of NMU in PTC cells, finding that the proliferative ability of PTC cells and the activity of KRAS pathway was significantly decreased after knockdown of the NMU gene. Our findings indicated that NMU was an important oncogene that affected PTC development via the KRAS signaling pathway.

## RESULTS

### Identification of DEGs in PTC

**Figure 1:**
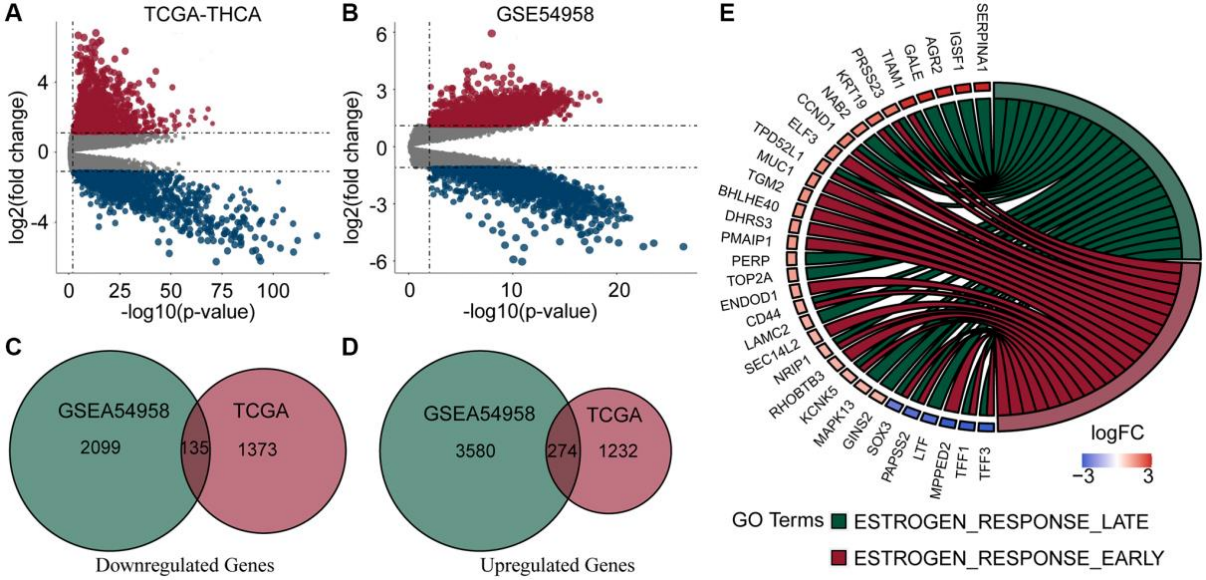
**Analysis of DEGs and enrichment in PTC and normal thyroid samples.** (**A**, **B**) Volcano plots of DEGs of TCGA-THCA and GSE54958 datasets. (**C**, **D**) Venn diagram of down- and up-regulated genes in TCGA and GSE54958 showed that 135 genes were down-regulated and 274 genes were upregulated. (**E**) DEGs were predominantly enriched in estrogen response-related pathways.

We downloaded complete clinical sequencing data for 501 thyroid cancer tissues and 58 normal tissues from the TCGA database. In addition, 25 TC tissues and 7 normal tissues were obtained from the GSE54958 dataset. Differential expression and Venn analyses identified 274 genes upregulated and 135 genes downregulated in both datasets (Figure 1A–1D). Finally, an enrichment analysis of these genes revealed that they were mainly enriched in estrogen response-related pathways (Figure 1E).

### Identification of independent prognostic factors in estrogen-related genes

**Figure 2:**
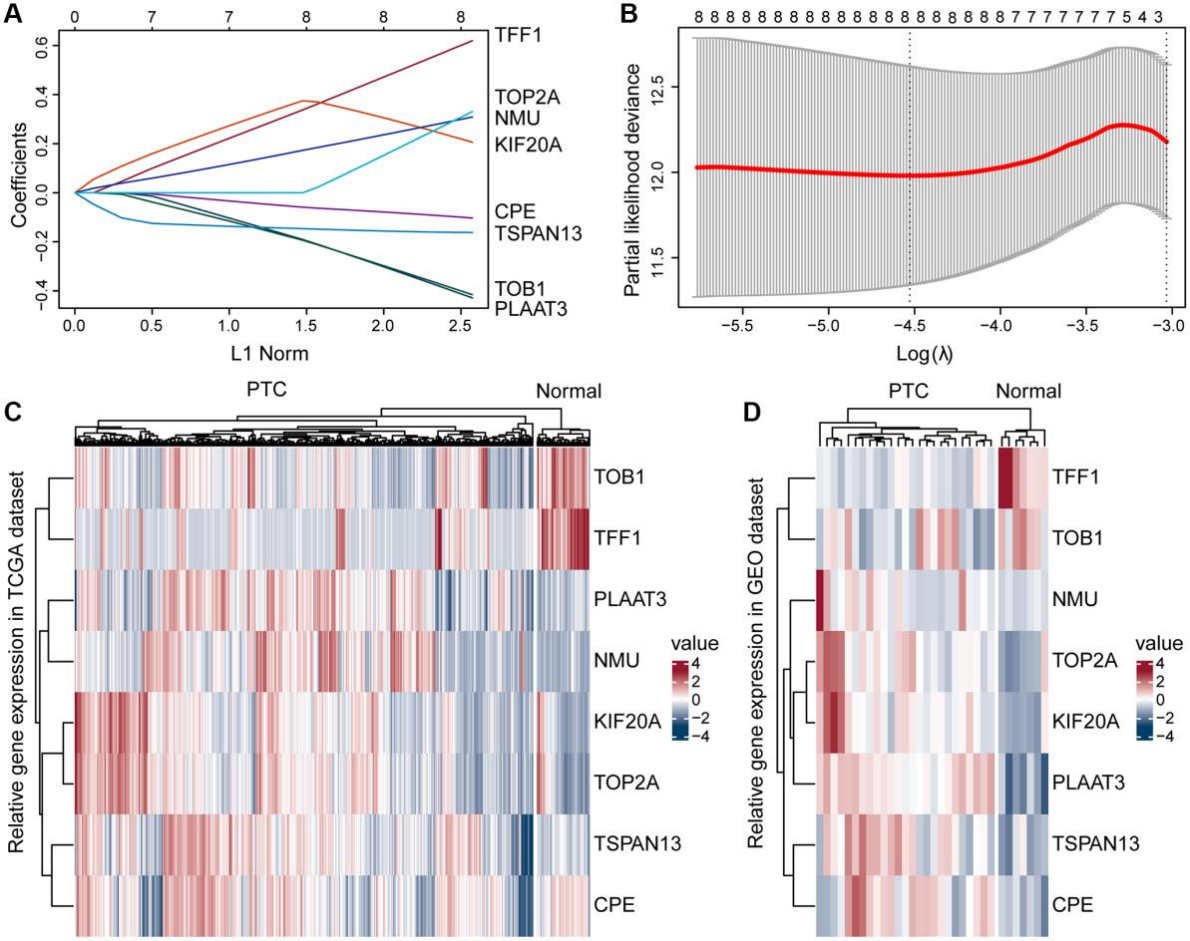
**Construction of the prognostic risk model.** (**A**) A Lasso coefficient profile for the prognostic value. (**B**) Partial likelihood distribution with the corresponding λ-logarithm value. (**C**, **D**) Based on the TCGA and GSE54958 datasets, ERGs were differentially expressed in the PTC and normal thyroid tissues, including TOB1, TFF1, PLAAT3, NMU, KIF20A, TOP2A, TSPAN13, and CPE.

To find independent prognostic factors, we downloaded ERGs sets (Supplementary Table 1) and filtered out duplicate and poorly expressed genes, leaving 299 genes for subsequent analysis (Supplementary Table 2). Univariate analysis of these 299 genes identified eight genes with *p* < 0.05 (Supplementary Table 2): CPE, KIF20A, NMU, PLAAT3, TFF1, TOB1, TOP2A, and TSPAN13. Then we further filtered the genes by Lasso cox analysis, and the 8 genes were retained, which we used to construct the risk score model, risk score = (0.33 × TFF1 expression) + (−0.47 × PLAAT3 expression) + (−0.43 × TOB1 expression) + (−0.48 × TSPAN13 expression) + (0.53 × KIF20A expression) + (−0.27 × CPE expression) + (0.19 × NMU expression) + (0.39 × TOP2A expression) (Figure 2A, 2B). Next, we created a heatmap based on their expression in the TCGA and GSE54958 datasets, showing that TOB1 and TFF1 were more highly expressed in normal thyroid tissues than in PTC tissues, but PLAAT3, NMU, KIF20A, TOP2A, TSPAN13, and CPE were more highly expressed in PTC tissues (Figure 2C, 2D).

### Validation of the estrogen-response related prognostic signature

**Figure 3:**
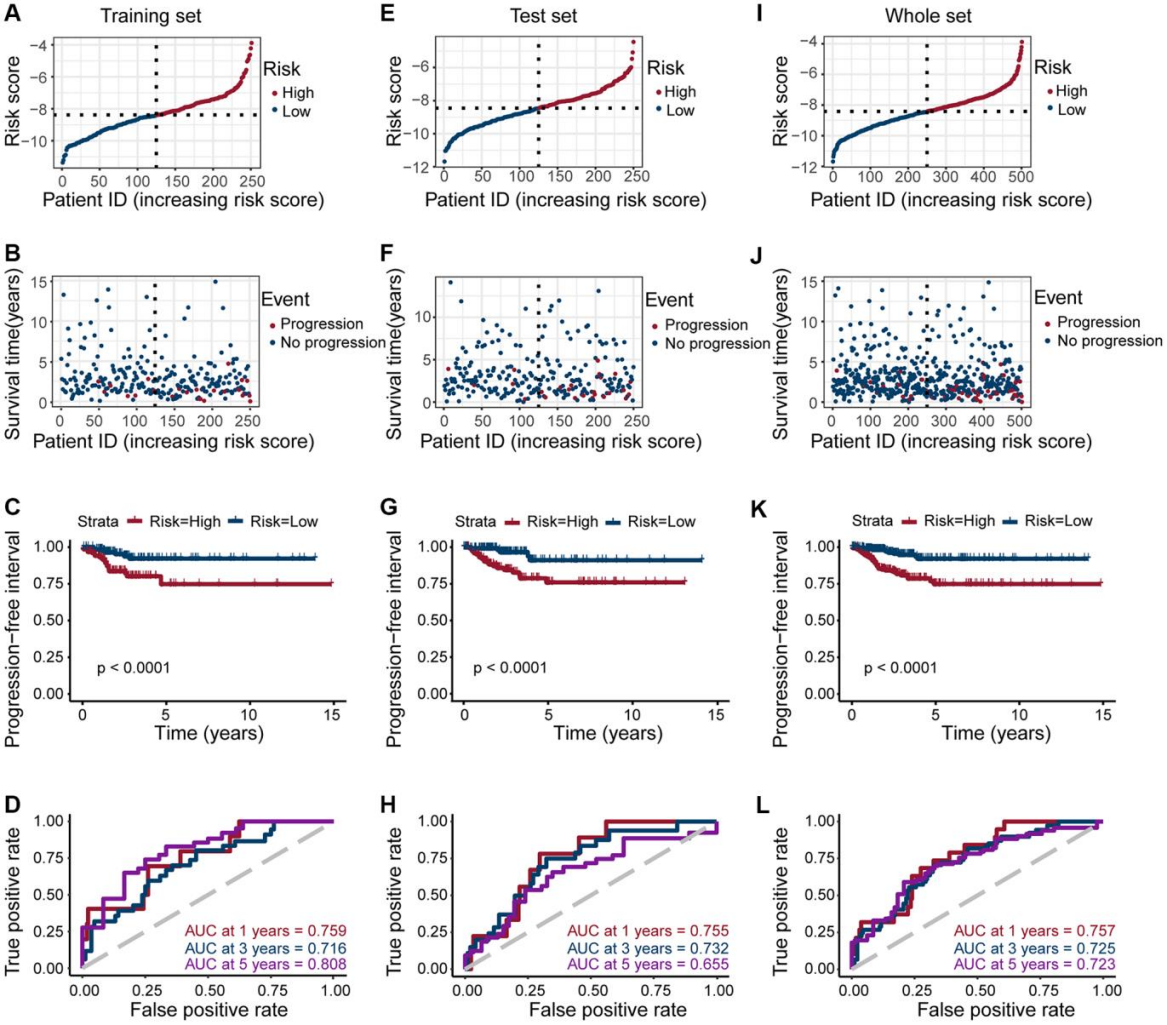
**Risk grouping and PFI prediction for three data sets (training set, test set, and whole set).** (**A**, **E**, **I**) Division of high-risk and low-risk groups in the training set, test set, and whole set (median value of risk scores for training set, test set, whole set = −8.388908, −8.454174, and −8.413054, respectively). (**B**, **F**, **J**) Survival time of patients in the progressive and non-progressive groups in the training set, test set and whole set. (**C**, **G**, **K**) Patients in the high-risk group had shorter PFI than in the low-risk group in the training set, test set and whole set (All *p* < 0.001). (**D**, **H**, **L**) The strong predictive potential of the prognostic features constructed from TOB1, TFF1, PLAAT3, NMU, KIF20A, TOP2A, TSPAN13, and CPE, similar results were observed in the test set and whole set.

To confirm the strong predictive potential of the prognostic features, we used the R *caret* package to evenly randomize the TCGA cohort into a training and test set (Supplementary Table 3). Next, we divided the patients into high- and low-risk groups based on the expression of eight ERGs in the training, test, and whole set, (median value of risk scores for training set, test set, whole set = −8.388908, −8.454174, and −8.413054, respectively) (Figure 3A, 3E and 3I), and observed the risk score distribution and the survival status of individual patients (Figure 3B, 3F and 3J).

Patients in the high-risk score group are more likely to progress, we observed a similar result across all datasets (all *p* < 0.0001; Figure 3C, 3G, and 3K). To further explore the accuracy of prognostic features in predicting patients’ PFI, we also performed a time-dependent ROC curve prediction. We found that the AUC for prognostic features in the training set reached 0.759, 0.716, and 0.808 at 1, 3, and 5 years, respectively. Similarly, in the test set, the AUC reached 0.755, 0.732, and 0.655, respectively. And in the whole set, the AUC reached 0.757, 0.725, and 0.723, respectively (Figure 3D, 3H, and 3L). Altogether, these results suggested that prognostic features of ERGs can predict PTC development.

### Relationship between risk grouping and clinicopathological characteristics of PTC

**Figure 4:**
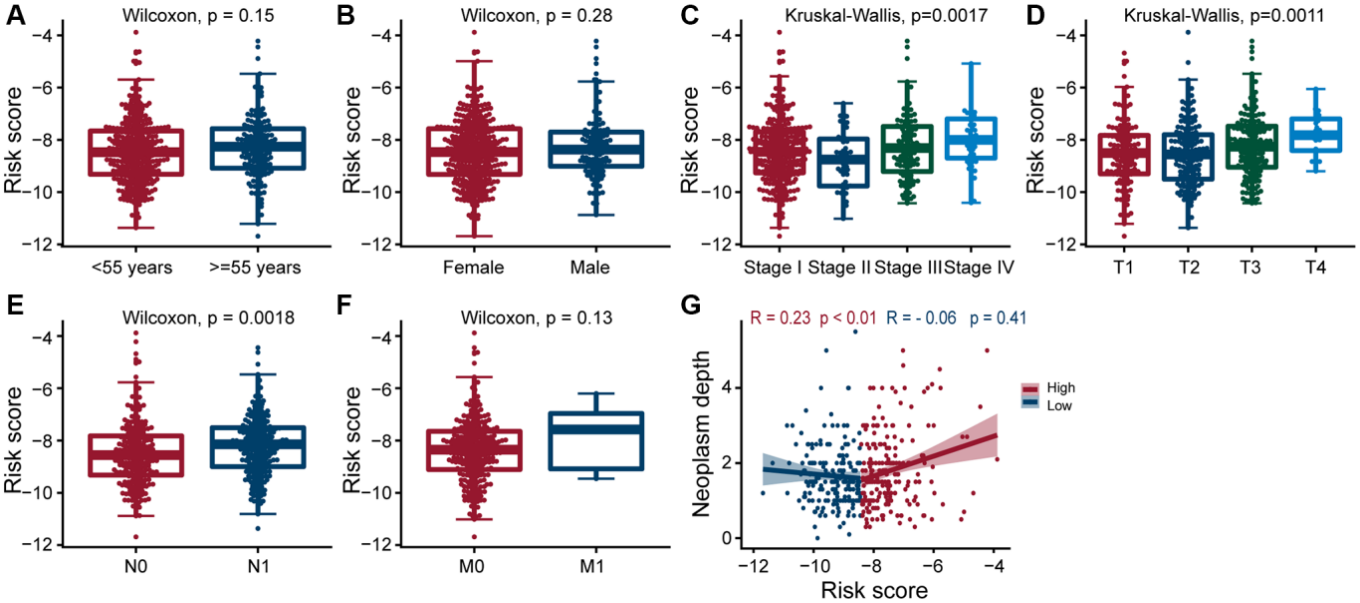
**Clinical evaluation of patients with PTC in different risk groups.** Clinical by the estrogen-related genes. Correlation analysis of risk score comprised of ERGs with age (**A**), gender (**B**), pathologic stage (**C**), T-stage (**D**), N-stage (**E**), M-stage (**F**), and neoplasm depth (**G**) in PTC patients. The risk score was significantly correlated with pathologic stage (*p* = 0.0017), T-stage (*p* = 0.0011), N-stage (*p* = 0.0018), and neoplasm depth (*p* < 0.01 and *p* = 0.41).

Next, we explored the clinical significance of risk score, and found that it was significantly positively correlated with pathologic stage, primary tumor size, regional lymph node metastasis, and tumor depth (All *p* < 0.05; Figure 4C–4E, 4G), but not with age, gender, and distant metastasis (Figure 4A, 4B and 4F). These results suggested that as the expression of ERGs increased, the likelihood of PTC progressing to advanced tumors also increased, and overall patient prognosis worsen.

### NMU identified as a hub gene affecting PTC prognosis

**Figure 5:**
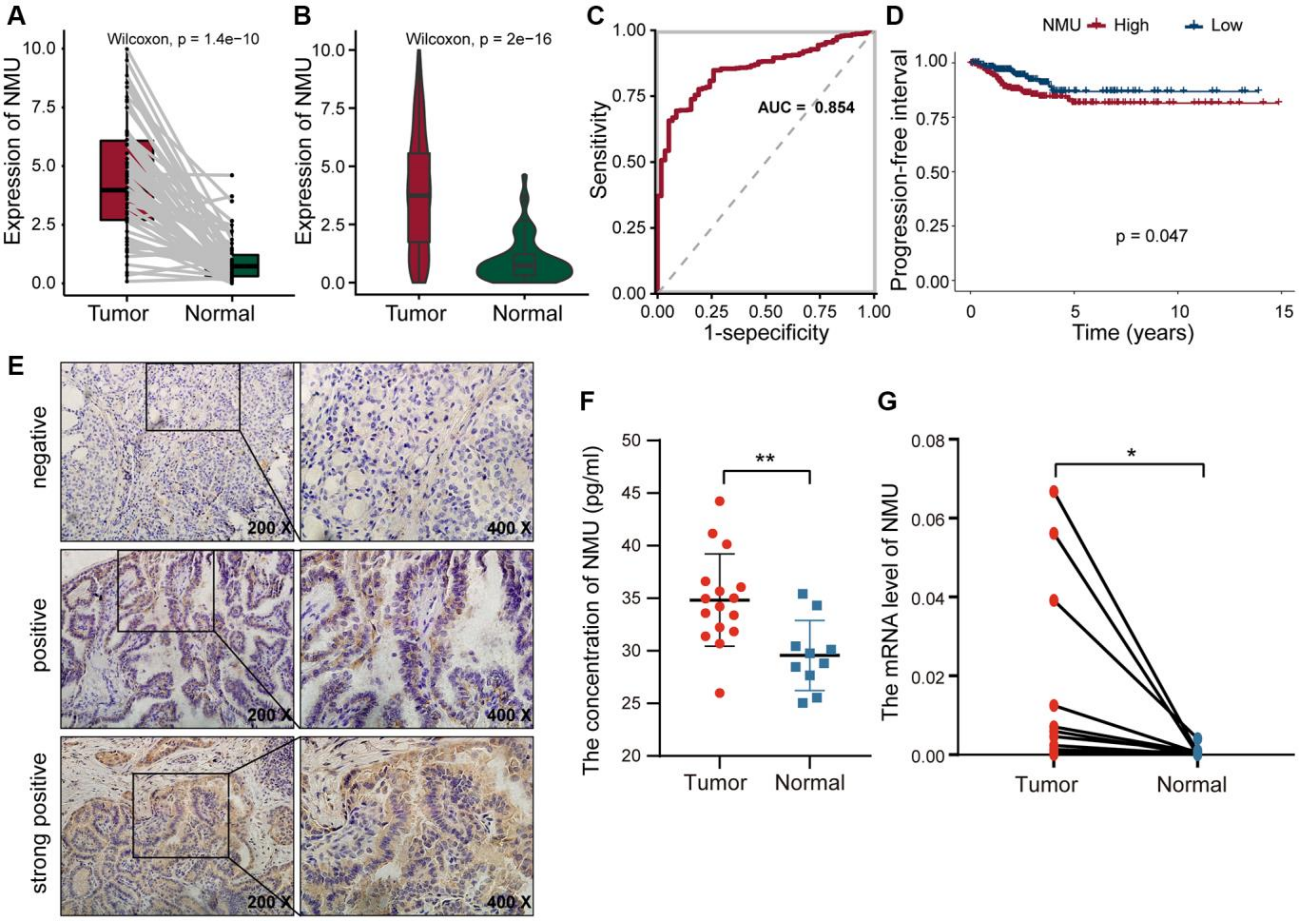
**The differential expression of NMU in tumor and normal tissues.** (**A**) Differential expression of NMU in tumor and normal tissues in the TCGA database (*p* = 1.4e-10). (**B**) Pair analysis of NMU expression between tumor and normal tissues from the same patient in the TCGA database (*p* = 2e-16). (**C**) The AUC of NMU expression was 0.854, obtained by a ROC curve analysis, distinguishing normal tissues from tumor tissues. (**D**) K-M analysis showed that patient prognosis was significantly worse when NMU expression was elevated (*p* = 0.047). (**E**) NMU was significantly overexpressed in tumor tissues, with a positive rate of 72% (18/25). (**F**) Serum NMU protein levels were significantly higher than in healthy individuals (^**^p < 0.01). (**G**) RT-qPCR confirmed that NMU expression was significantly increased in tumor tissues (^*^p < 0.05).

We performed K-M analysis for each of the above eight genes and could found that higher the expression of TOB1, NMU, and TOP2A, the worse the prognosis of PTC patients (Supplementary Figure 1). Since NMU has been previously found to be highly expressed in PTC cells [24], and we hypothesized that NMU was a potential biomarker for PTC. In the TCGA dataset, we found overall NMU expression to be significantly higher in tumor tissues than in normal tissues (*p* = 1.4 × 10^−10^; Figure 5A). Pair analysis also found that NMU expression was significantly higher in tumor tissues from the same patients (*p* = 2 × 10^−16^; Figure 5B). In ROC curve analysis of NMU expression, the AUC was 0.854, distinguishing normal and tumor tissues (Figure 5C). K-M analysis showed that the prognosis of patients was significantly worse with increased NMU expression (*p* = 0.047; Figure 5D). Altogether, these results suggested that NMU was a poor biomarker for predicting PTC patient prognosis.

### NMU may play an important oncogenic role in PTC

To confirm NMU expression levels, we quantified NMU protein levels in tumor tissues from 25 PTC patients with IHC, and found that the NMU positive rate was as high as 72% (18/25). NMU, as a secreted protein, was mainly present in the cytoplasm as brown and yellow granules (Figure 5E). The serum NMU levels in PTC patients (25.99–44.23 pg/ml; median = 34.82 pg/ml) was significantly higher than in healthy controls individuals (median = 29.55 pg/ml; 25.03–35.41 pg/ml; *p* < 0.01; Figure 5F). Finally, we compared NMU expression in 16 randomly selected pairs of cancerous and neighboring normal tissues from PTC patients, finding that NMU expression was significantly higher in cancerous tissues than in normal tissues (*p* < 0.05; Figure 5G). Therefore, these results suggested that NMU expression was elevated in PTC patients, consistent with our analysis results.

**Figure 6:**
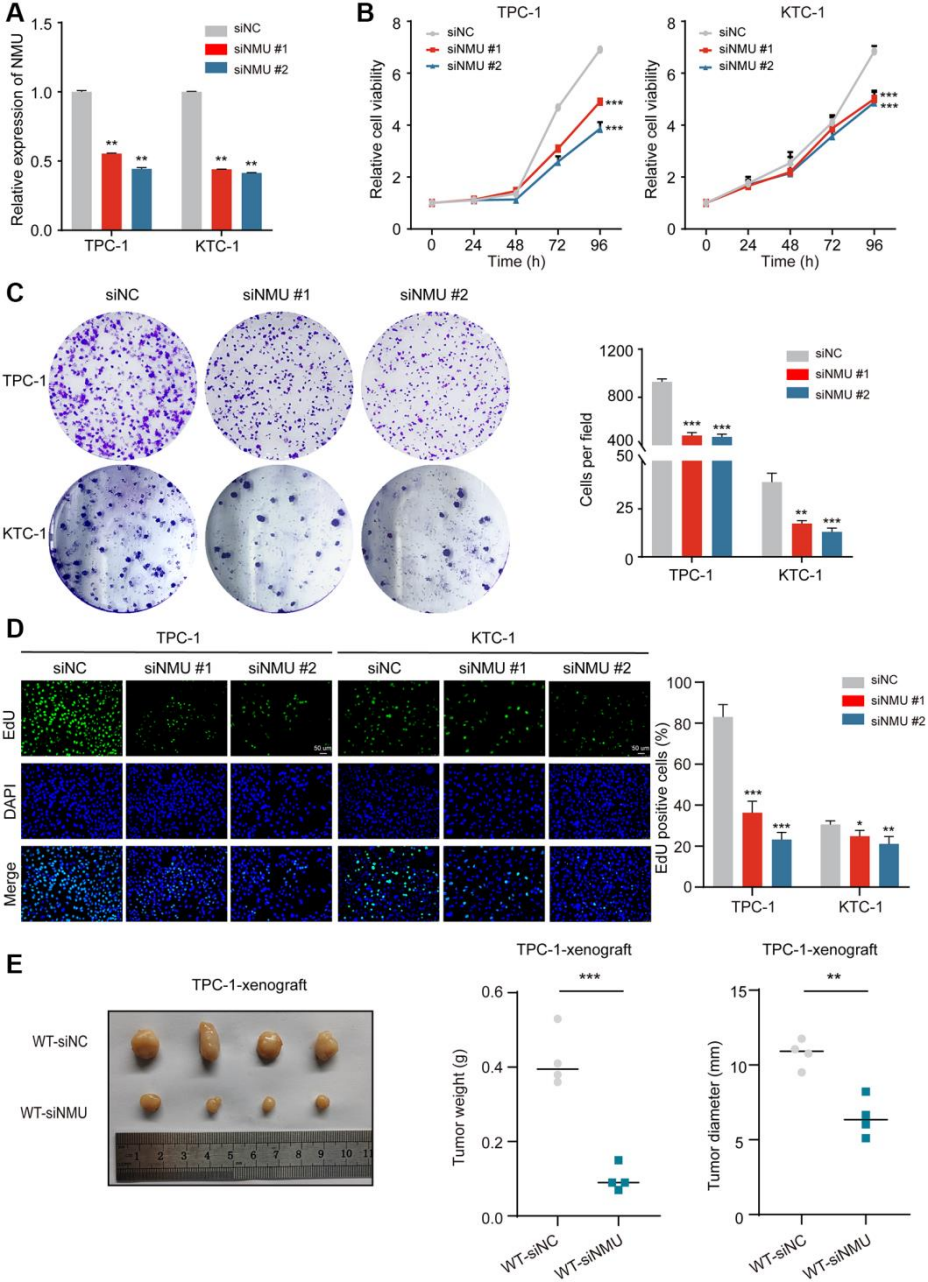
**NMU promotes PTC cell viability, colony formation, cell proliferation abilities and tumor growth.** (**A**) Suppression of NMU by NMU-siRNAs (siNMU#1 and siNMU#2) determined by RT-qPCR. (**B**) NMU-siRNAs significantly reduced cell viability of TPC-1 and KTC-1 cells compared to control group. (**C**) NMU-siRNAs significantly reduced colony formation number in TPC-1 and KTC-1 cells. (**D**) The inhibitory effect of NMU-siRNAs on cell proliferation was confirmed with the EdU assay (^*^p < 0.05; ^**^p < 0.01; ^***^p < 0.001). (**E**) Subcutaneous xenograft tumors formed by TPC-1 cells in nude mice. siRNA transfection was adopted to reduce NMU levels in tumors. Tumor weights and tumor diameter were measured for each group (*n* = 4 per group). NMU-siRNAs significantly inhibited the growth of tumors in nude mice. (^**^p < 0.01; ^***^p < 0.001).

To determine the effect of NMU knockdown on cell proliferation, we used TPC-1 and KTC-1 cells and confirmed they had significant knockdown efficiency (*p* < 0.01; Figure 6A), then performed CCK-8, clone formation assays and EdU proliferation. We found that NMU knockdown significantly reduced the activity, colony formation ability and proliferation ability of TPC-1 and KTC-1 cells (^*^p < 0.05; ^**^p < 0.01; ^***^p < 0.001; Figure 6B–6D). And it was further confirmed by *in vivo* experiments that NMU knockdown could significantly inhibit the growth of PTC (^**^p < 0.01; ^***^p < 0.001; Figure 6E).

Therefore, these results suggested that NMU expression may promote malignant PTC progression and cell growth.

### NMU may exert oncogenic effects by affecting KRAS signaling pathway in PTC

**Figure 7:**
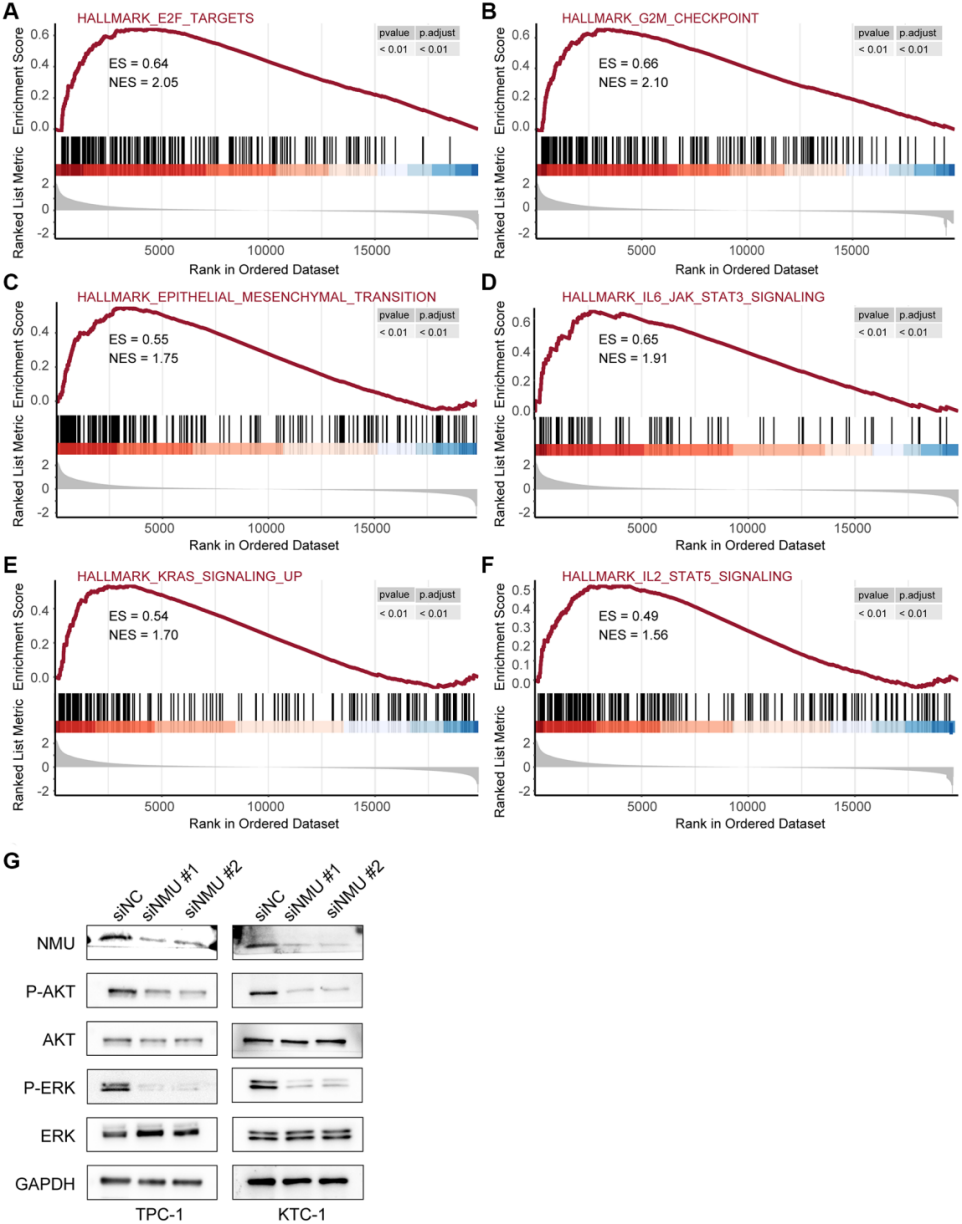
**The top 6 significantly enriched pathways in the high-risk and low-risk groups.** GSEA identified six pathways significantly enriched in the high-risk group, including E2F target (**A**), G2M checkpoint (**B**), epithelial-mesenchymal transition (**C**), IL6/JAK/STAT3 signaling (**D**), KRAS signaling (**E**), and IL2/STAT5 signaling (**F**) (All *p* < 0.01). (**G**) Western blots of KRAS signaling in TPC-1 and KTC-1 cells with and without NMU knockdown. GAPDH was used as the loading control, p-AKT and p-ERK levels were significantly lower with NMU knockdown.

Next, we further examined the pathways significantly enriched in the high-risk group, identifying six signaling pathways: E2F targets, G2M checkpoint, epithelial-mesenchymal transition, IL6/JAK/STAT3 signaling, KRAS signaling, and IL2/STAT5 signaling (All *p* < 0.01; Figure 7A–7F), indicating that NMU may promote malignant PTC progression via these signaling pathways.

To confirm whether NMU in PTC affects the KRAS signaling pathway, we first confirmed that NMU had significant knockdown efficiency in TPC-1 and KTC-1 cell lines by western blot. Then, we examined the classical markers of KRAS signaling pathway, including AKT, p-AKT, ERK, and p-ERK. We found that p-AKT and p-ERK had significantly lower expression with NMU knockdown, suggesting that decreased NMU expression may inhibit malignant PTC progression by reducing the phosphorylation level of the KRAS signaling pathway (Figure 7G). Altogether, these results suggested that NMU is critical for PTC tumorigenicity and may contribute to PTC development via activating the KRAS signaling pathway.

## DISCUSSION

PTC is the most common type of TC pathology with a better prognosis. However, many PTC patients can still develop resistance to conventional treatment, develop local and distant metastasis and have a high probability of recurrence [25]. PTC occurs most often in women, and its incidence rate is three times than that of men [26, 27]. It suggests that PTC development may be closely related to estrogen levels. Therefore, it is essential to identify the targets of estrogen action and the molecular mechanisms in PTC.

Based on bioinformatics analysis, we identified DEGs between PTC with normal thyroid tissue enriched for ERGs, developed a risk-prognosis model, and created a heatmap of the ERGs expression. This heatmap contained eight genes: TOB1, TFF1, PLAAT3, NMU, KIF2OA, TOP2A, TSPAN13, and CPE. TOB1 is a tumor suppressor gene and a key determinant of survival in estrogen-dependent ER-positive breast cells [28]. TFF1 is a well-known tumor suppressor in gastric cancer (GC) and a downstream target of the nuclear receptor estrogen-related receptor gamma [29]. Higher TOP2A expression was significantly associated with tumor size, poor grading, human epidermal growth factor receptor 2 expression, and positive lymph nodes in breast cancer [30]. However, since the function and mechanism of ERGs in PTC risk-prognosis models have not been reported, their roles need to be further explored.

This study focused on the association between NMU and PTC prognosis based on the results of univariate and Lasso Cox regression analysis. We hypothesize that NMU may help facilitate the progress of PTC. *In vivo* experiments have shown that NMU expression is higher in the tissues and serum of PTC patients compared to healthy individuals, and *in vitro* experiments have also shown that the proliferation and colony formation ability of PTC cells are significantly reduced with NMU knockdown.

NMU expression has been found to be elevated in various cancers, including hepatocellular cancer, pancreatic cancer, and colorectal cancer (CRC) [22, 23, 26]. It has been reported that NMU accelerates the migration and invasion of CRC cells by activating ERK1/2 kinases [22]. In this study, we hypothesized that NMU might promote malignant PTC progression through E2F targets, G2M checkpoints, epithelial-mesenchymal transition, the IL6/JAK/STAT3 signaling pathway, the KRAS signaling pathway, and the IL2/STAT5 signaling pathway. In addition, we experimentally confirmed that NMU might promote malignant PTC progression by activating the KRAS signaling pathway. Previous studies have found that NMU expression correlates with prognosis in PTC via bioinformatics analysis. However, none have performed biological experiments to support this conclusion [31–33]. Therefore, this study was the first to perform biological experiments on NMU to support their conclusion.

However, there were some limitations to this study. Firstly, its model was constructed and validated using data from public TCGA and GEO databases, and more prospective data were needed to validate the clinical applicability of our model. Secondly, we confirmed NMU expression in PTC and its role in promoting PTC proliferation without explored its other functions. Finally, we screened for NMU from ERGs, but we have not performed further clinical studies to confirm that estrogen levels influence its expression in PTC patients. Therefore, while it is clear that NMU can promote PTC progression, further studies are required to determine whether it is a significant prognostic biomarker for PTC.

This study first identified various DEGs using PTC data from the public TCGA and GEO databases, found them predominantly enriched in estrogen response-related pathways. Eight hub genes that could be potential PTC biomarkers were identified via risk models. NMU was chosen as the focus of this study based on the differential expression of eight genes in PTC and normal tissues and the impact on the PFI, and confirmed its expression in PTC and its important role in promoting PTC progression, showing that NMU might be an important indicator for PTC diagnosis and prognosis. In conclusion, our finding is important for elucidating the potential molecular biological mechanisms of PTC and developing new prognostic biomarkers.

## MATERIALS AND METHODS

### Data collection and preprocessing

Row count and normalized RNA-seq data and corresponding clinical features of PTC patients were downloaded from the UCSC Xena browser (https://xena.ucsc.edu/). The GSE54958 microarray dataset was downloaded from the GEO database (https://www.ncbi.nlm.nih.gov/geo/). We also downloaded the set of genes associated with the HALLMARK_ESTROGEN_RESPONSE_EARLY and HALLMARK_ESTROGEN_RESPONSE_LATE pathways from the MSigdb (http://www.gsea-msigdb.org/gsea/msigdb/collections.jsp), and filtered out duplicates and poorly expressed genes.

### Identification of DEGs between normal and tumor tissues in PTC

We used the limma package in the R statistical software to identify DEGs for the GEO and TCGA expression data [34] with a log_2_ (fold change) threshold of >1.1 and a significance threshold of *p* < 0.01. Genes that were jointly up- or down-regulated in both datasets were considered DEGs. In addition, we used the enricher function of the R clusterProfiler package to calculate the enrichment of the hallmark gene sets in the MSigdb [35].

### Construction and validation of the prognostic model

The whole set with complete clinical information were equally divided into training and test sets using the R package caret. The R survival package was used to perform univariate Cox regression analysis, and the R glmnet package was used to perform lasso Cox regression analysis. The estrogen response-related DEGs with a significant prognostic value (*p* < 0.05) were screened. We constructed a risk model based on the screened prognostic genes, risk score = (coefficient gene 1 × gene 1 expression) + (coefficient gene 2 × gene 2 expression) + (coefficient gene *N* × gene *N* expression), where the coefficient was from the univariate Cox regression analysis of the training set. Patients were divided into a high-risk score group and a low-risk score group, depending on the median value of the risk score. The difference in survival between the two groups was assessed with Kaplan-Meier (K-M) survival curves and compared by the log-rank test. Time-dependent ROC curves for PFI of 1, 3, and 5 years were constructed, and AUC values were calculated to assess the accuracy of the prognostic model. Then a heatmap was also created based on the expression of eight genes in the TCGA and GSE54958 datasets between the PTC and normal tissues.

### Correlation between the risk scores and clinical indicators

The “beeswarm” R package was used to demonstrate the correlation between risk scores and clinical features (including age, gender, TNM stage, and pathologic stage), and the effect of high- and low-risk groups on neoplasm depth, to better assess the impact of different clinical features on PTC prognosis. The statistical significance was analyzed by the Kruskal-Wallis test.

### Differential expression and survival analysis of NMU in tumor and normal tissues

We performed K-M analysis on each of the above eight genes to assess the correlation between the expression of these genes and the prognosis of PTC patients. Differential expression of NMU in PTC and normal tissues and NMU in Pair-PTC tissues were evaluated through the TCGA database. Accuracy of differential expression of NMU in PTC and normal tissue was assessed by ROC curves. The difference in PFI between the NMU high expression group and NMU low expression group was assessed with K-M survival curves and compared by the log-rank test.

### Gene set enrichment analysis

GSEA is a computational method to determine whether a pre-defined set of genes shows significant differences between two biological states. We performed the GSEA for the Hallmark gene sets in the high-risk group and the low-risk group with the R clusterProfiler package.

### Patient samples

From 2020 to 2021, a total of 16 PTC tissues and 16 paired non-tumors thyroid samples were collected for RT-qPCR, and 25 paraffin tissues were collected for IHC from Tianjin Medical University Cancer Institute and Hospital (Tianjin, China). The peripheral blood of 16 PTC patients were collected to quantify NMU levels by ELISA, and 10 healthy individuals were collected as controls. This study was approved by the Ethics Committee of the Tianjin Medical University Cancer Institute and Hospital. All procedures were performed according to the Declaration of Helsinki and relevant Chinese policies.

### IHC staining

IHC staining was performed on deparaffinized tissue sections of human PTC surgical specimens. Rehydration was performed with an ethanol series, and sections were blocked with 5% normal goat serum and 3% bovine serum albumin in tris-buffered saline for 60 min. Sections were incubated with 3% hydrogen peroxide for 15 min at room temperature to block endogenous peroxidase and then incubated overnight at 4°C with the appropriate primary antibody. Horseradish peroxidase conjugated antibodies were detected by 3,3′-diaminobenzidine IHC staining.

### Cell culture and cell transfection

The cell lines for PTC, TPC-1, and KTC-1 cells were cultured in Roswell Park Memorial Institute (RPMI)-1640 medium (Gibco; Gaithersburg, MD, USA) supplemented with 10% fetal bovine serum (FBS; Biological Industries; Cromwell, CT, USA), 2 mM L-glutamine (Gibco), penicillin, and streptomycin (Gibco). Cells were maintained in a humidified incubator at 37°C with 5% carbon dioxide (CO_2_). We synthesized a small interfering RNA (siRNA) against NMU (Qingke Bio; Beijing, China) to study NMU function with the sequence shown in Supplementary Table 4. TPC-1 and KTC-1 cells were transfected using Lipofectamine 2000 (Invitrogen; Waltham, MA, USA) according to the manufacturer’s recommended protocol, which used serum-free medium for transfection that was replaced with complete medium containing 10% FBS after 6 h. Cells were harvested for subsequent experiments after continued incubation for 24–48 h.

### RNA isolation and RT-qPCR

Total RNA was extracted with TRIzol reagent (AC0101-B; SparkJade, China) added to 25 pairs of PTC tissues and their corresponding paracancerous tissues. For RT-qPCR, PCR reactions were performed with 2 × HQ SYBR qPCR Mix (ZF501; ZOMANBIO; Beijing, China) on an Applied Biosystems 7500 Fast Real-Time PCR System (Foster City, CA, USA) according to the manufacturer’s instructions. The primers used in this study were listed in Supplementary Table 5.

### Cell proliferation and colony formation assay

Cell proliferation assays were performed using Cell Counting Kit-8 (CCK-8; C6005M; US Everbright; Silicon Valley, CA, USA) and 5-ethynyl-2′-deoxyuridine (EdU; C6015M; US Everbright) kits according to their manufacturer’s instructions. For the cell viability assay, PTC cells were inoculated into 96-well plates at a density of 1,000 cells/well and left to stand with 10 μL of CCK-8 reagent for 2.5 h at 37°C with 5% CO_2_, then incubated for 0, 24, 48, 72 and 96 h following transfection. For the EdU assay, 5 × 10^4^ cells were inoculated into a 24-well plate and cultured in complete culture medium. After 24 h, cells were stained and photographed according to the manufacturer’s instructions. For the colony formation assay, the corresponding siRNA was transfected into TPC-1 and KTC-1 cells for 24 h. Then, cells were digested with trypsin and transferred into six-well plates (1000 cells/well) and kept for two weeks for colony formation assays. All experiments were repeated at least three times.

### Western blotting and antibodies

Total protein was extracted from harvested cells using radioimmunoprecipitation assay lysis buffer (R0020; Solarbio; Shanghai, China). Protein samples were subjected to 10% sodium dodecyl sulfate (SDS)-polyacrylamide gel electrophoresis and transferred onto a polyvinylidene difluoride membrane, and then incubated with a specific antibody at 4°C overnight: NMU (24862-1-AP; Proteintech; Rosemont, IL, USA), protein kinase B (AKT; 4685S; Cell Signaling Technology [CST]; Danvers, MA, USA), phosphorylated AKT (p-AKT; 4060S; CST), extracellular signal-regulated kinase (ERK; 4695S; CST), and phosphorylated ERK (p-ERK; 4370S; CST). Then, the PVDF membranes were incubated with secondary antibodies, including goat anti-rabbit immunoglobulin G (IgG[H+L])-HRP (UT2001; UTIBODY; Tianjin, China) at room temperature for 1 h. An antibody against glyceraldehyde 3-phosphate dehydrogenase (GAPDH) was used as the control (R5174; CST).

### *In vivo* studies

Four-week-old female BALB/c nude mice were purchased from the GemPharmatech Co., Ltd (Jiangsu, China). A PTC subcutaneous tumor model was constructed using TPC-1 cells. When the tumor diameter reached ~3 mm, mice were randomly divided into two groups: (1) NMU down-regulation group and (2) control group. NMU down-regulation was performed by siRNA transfection (Qingke Bio; Beijing, China). Each tumor was locally injected with 5 nmol/20 g of siRNA twice a week for 2 weeks. The animals were killed 35 days after injection, and the tumors were collected to measure diameter and weight. All animal experiments were performed humanely according to the guidelines reviewed by the Animal Ethics Committee of the Tianjin Medical University Cancer Institute and Hospital.

### Statistical analysis

Data analysis was performed by using GraphPad Prism 9.0 (San Diego, CA, USA). The two-sided Student’s *t*-test was used to compare unpaired data. PFI analyses were performed with K-M curves and log-rank tests. Spearman rank correlation coefficient (ρ) was used to assess the correlation between risk score and neoplasm depth. The Cox hazard regression model was used for univariate analysis, expressed as HR with the 95% CI. *P* value < 0.05 was considered statistically significant.

## Supplementary Materials

Supplementary Figure 1

Supplementary Table 1

Supplementary Table 2

Supplementary Tables 3-5
