# Supplementary material for: Identification and validation of eight estrogen-related genes for predicting prognosis of papillary thyroid cancer
Source: Aging (Albany NY). 2023 Mar 13;15(5):1668–84. doi: 10.18632/aging.204582 (PMC10042678; doi:10.18632/aging.204582)
Supplement: Supplementary Figure 1 [file aging-15-204582-s001.pdf]

SUPPLEMENTARY FIGURE

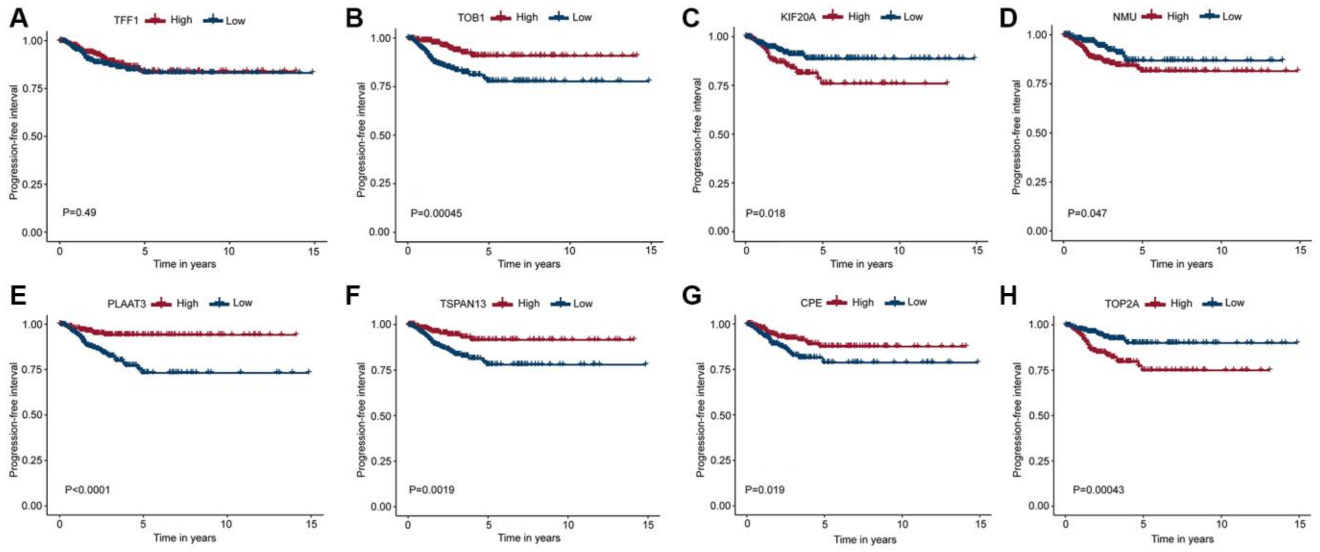

Supplementary Figure 1. The K-M survival analysis of 8 hub genes in TCGA-THCA. The K-M survival analysis of TFF1 (**A**), TOB1 (**B**), KIF20A (**C**), NMU (**D**), PLAAT3 (**E**), TSPAN13 (**F**), CPE (**G**) and TOP2A (**H**) in TCGA-THCA. The expression of TFF1 was not significantly correlated with the prognosis of PTC patients, the expression of TOB1, PLAAT3, TSPAN13 and CPE was significantly positively correlated with the prognosis of PTC patients (All $p < 0.05$), and the expression of KIF20A, NMU and TOP2A was significantly negatively correlated with the prognosis of PTC patients (All $p < 0.05$).
