# Supplementary material for: Identification and validation of eight estrogen-related genes for predicting prognosis of papillary thyroid cancer
Source: Aging (Albany NY). 2023 Mar 13;15(5):1668–84. doi: 10.18632/aging.204582 (PMC10042678; doi:10.18632/aging.204582)
Supplement: Supplementary Table 1 [file aging-15-204582-s002.docx]

**Supplementary Table 1. The estrogen-related genes (ERGs) in PTC.**

| **Term** | HALLMARK_ESTROGEN_RESPONSE_EARLY | HALLMARK_ESTROGEN_RESPONSE_LATE |
| --- | --- | --- |
| **Gene** | GREB1 | TFF1 |
|  | CA12 | SLC9A3R1 |
|  | SLC9A3R1 | TPD52L1 |
|  | MYB | PRSS23 |
|  | ANXA9 | CA12 |
|  | IGFBP4 | PDZK1 |
|  | SYBU | ANXA9 |
|  | NPY1R | CELSR2 |
|  | PDZK1 | TJP3 |
|  | NRIP1 | PGR |
|  | MLPH | RET |
|  | HSPB8 | MYB |
|  | EGR3 | TPBG |
|  | KRT19 | EGR3 |
|  | LRIG1 | ARL3 |
|  | KDM4B | OLFM1 |
|  | PGR | NPY1R |
|  | RHOBTB3 | SCNN1A |
|  | TPD52L1 | XBP1 |
|  | ELOVL2 | AREG |
|  | RET | IL17RB |
|  | TPBG | NRIP1 |
|  | TFF1 | ASS1 |
|  | MAPT | TFF3 |
|  | SCNN1A | FKBP4 |
|  | ABAT | SLC27A2 |
|  | FLNB | SEMA3B |
|  | XBP1 | GPER1 |
|  | CELSR2 | LLGL2 |
|  | RAB31 | AGR2 |
|  | MYBL1 | KRT19 |
|  | MREG | CCN5 |
|  | FAM102A | BLVRB |
|  | MSMB | FLNB |
|  | STC2 | PDCD4 |
|  | RETREG1 | CALCR |
|  | SIAH2 | IGFBP4 |
|  | ZNF185 | DNAJC12 |
|  | SLC19A2 | TIAM1 |
|  | SLC1A4 | TSPAN13 |
|  | FHL2 | CXCL12 |
|  | BCL2 | RAB31 |
|  | PMAIP1 | PKP3 |
|  | AREG | CYP26B1 |
|  | OVOL2 | FKBP5 |
|  | TSKU | SIAH2 |
|  | ADCY9 | ISG20 |
|  | RASGRP1 | TMPRSS3 |
|  | MUC1 | SERPINA3 |
|  | KAZN | WFS1 |
|  | SLC27A2 | MAPT |
|  | FKBP4 | PDLIM3 |
|  | CXCL12 | RBBP8 |
|  | TMPRSS3 | GJB3 |
|  | RARA | PRLR |
|  | IL17RB | SLC1A4 |
|  | CBFA2T3 | FOS |
|  | TFF3 | PLAAT3 |
|  | UGCG | SLC7A5 |
|  | CCND1 | SERPINA5 |
|  | SLC22A5 | IMPA2 |
|  | WFS1 | DHCR7 |
|  | PTGES | MYOF |
|  | WWC1 | CDH1 |
|  | CCN5 | EMP2 |
|  | MYC | OVOL2 |
|  | ITPK1 | DLG5 |
|  | TMEM164 | SOX3 |
|  | ARL3 | CHPT1 |
|  | MED13L | KLK10 |
|  | SEMA3B | ELOVL5 |
|  | KRT18 | RAPGEFL1 |
|  | SLC16A1 | JAK2 |
|  | TJP3 | SLC26A2 |
|  | SLC26A2 | SLC22A5 |
|  | FCMR | ITPK1 |
|  | SULT2B1 | PCP4 |
|  | SNX24 | PAPSS2 |
|  | TFAP2C | NAB2 |
|  | TTC39A | FAM102A |
|  | GJA1 | BCL2 |
|  | PRSS23 | LSR |
|  | OLFM1 | CACNA2D2 |
|  | RAPGEFL1 | CA2 |
|  | ASB13 | ASCL1 |
|  | TIPARP | ACOX2 |
|  | ABCA3 | CISH |
|  | FRK | GLA |
|  | DHRS2 | PTGES |
|  | AQP3 | PERP |
|  | KCNK15 | OPN3 |
|  | TGIF2 | KRT13 |
|  | FOXC1 | HSPB8 |
|  | ELF3 | UGDH |
|  | REEP1 | CLIC3 |
|  | PEX11A | KLK11 |
|  | PODXL | PLAC1 |
|  | KLF4 | ABHD2 |
|  | BAG1 | SCARB1 |
|  | CELSR1 | DCXR |
|  | PLAAT3 | CCND1 |
|  | SLC7A5 | SFN |
|  | MPPED2 | ABCA3 |
|  | TIAM1 | SULT2B1 |
|  | CLDN7 | CCNA1 |
|  | MYOF | STIL |
|  | RBBP8 | MICB |
|  | OLFML3 | ZFP36 |
|  | GFRA1 | CAV1 |
|  | FARP1 | NBL1 |
|  | SVIL | CD44 |
|  | TGM2 | HR |
|  | DEPTOR | HOMER2 |
|  | CYP26B1 | BTG3 |
|  | PAPSS2 | GAL |
|  | SLC1A1 | ETFB |
|  | DLC1 | BAG1 |
|  | JAK2 | FRK |
|  | AFF1 | SLC16A1 |
|  | KLK10 | AFF1 |
|  | P2RY2 | TFAP2C |
|  | BLVRB | IGSF1 |
|  | CISH | HPRT1 |
|  | GLA | CDC6 |
|  | ADD3 | FARP1 |
|  | PDLIM3 | AMFR |
|  | MINDY1 | DHRS2 |
|  | FOS | NXT1 |
|  | KRT8 | S100A9 |
|  | SLC37A1 | SLC29A1 |
|  | B4GALT1 | SLC24A3 |
|  | CALCR | FOXC1 |
|  | ESRP2 | KIF20A |
|  | IGF1R | TOB1 |
|  | NBL1 | FDFT1 |
|  | SFN | DNAJC1 |
|  | OPN3 | TPSAB1 |
|  | ABHD2 | TSTA3 |
|  | AR | FGFR3 |
|  | SLC39A6 | SGK1 |
|  | SYT12 | ID2 |
|  | CD44 | GALE |
|  | MED24 | BATF |
|  | BCL11B | MAPK13 |
|  | CANT1 | FABP5 |
|  | KRT13 | MEST |
|  | KRT15 | JAK1 |
|  | TOB1 | CYP4F11 |
|  | SLC7A2 | KCNK5 |
|  | LAD1 | CPE |
|  | TUBB2B | XRCC3 |
|  | TBC1D30 | CXCL14 |
|  | SEC14L2 | SCUBE2 |
|  | ENDOD1 | CDC20 |
|  | HR | IL6ST |
|  | SCARB1 | GINS2 |
|  | NCOR2 | TRIM29 |
|  | RHOD | UNC13B |
|  | INPP5F | LAMC2 |
|  | PPIF | LARGE1 |
|  | DHRS3 | SLC2A8 |
|  | FDFT1 | PLXNB1 |
|  | GAB2 | PRKAR2B |
|  | UNC119 | RPS6KA2 |
|  | KLF10 | HSPA4L |
|  | HES1 | TFPI2 |
|  | FKBP5 | SERPINA1 |
|  | SLC2A1 | TNNC1 |
|  | AMFR | HMGCS2 |
|  | NADSYN1 | ALDH3A2 |
|  | INHBB | CD9 |
|  | BHLHE40 | IDH2 |
|  | CALB2 | SORD |
|  | FASN | MDK |
|  | CHPT1 | ALDH3B1 |
|  | MYBBP1A | PTGER3 |
|  | ELOVL5 | RABEP1 |
|  | DYNLT3 | KLF4 |
|  | ABLIM1 | PPIF |
|  | SOX3 | SNX10 |
|  | SLC24A3 | METTL3 |
|  | RAB17 | PLK4 |
|  | MAST4 | COX6C |
|  | KCNK5 | ST14 |
|  | ELF1 | NCOR2 |
|  | RPS6KA2 | MOCS2 |
|  | ISG20L2 | NMU |
|  | IL6ST | TH |
|  | SYNGR1 | RNASEH2A |
|  | SH3BP5 | CHST8 |
|  | ALDH3B1 | TST |
|  | THSD4 | TOP2A |
|  | CLIC3 | CKB |
|  | NXT1 | LTF |
|  | NAV2 | DUSP2 |
|  | RRP12 | PTPN6 |
|  | ADCY1 | ATP2B4 |
|  | DHCR7 | ST6GALNAC2 |
|  | MICB | ADD3 |
|  | AKAP1 | DYNLT3 |
