# Supplementary material for: Identification and validation of eight estrogen-related genes for predicting prognosis of papillary thyroid cancer
Source: Aging (Albany NY). 2023 Mar 13;15(5):1668–84. doi: 10.18632/aging.204582 (PMC10042678; doi:10.18632/aging.204582)
Supplement: Supplementary Table 2 [file aging-15-204582-s003.docx]

**Supplementary Table 2. The 299 estrogen-related genes (ERGs) involved in the univariate analysis.**

| **Genes** | **Beta** | **HR (95% CI)** | **HR** | **HR.confint.lower** | | **HR.confint.upper** | | **wald.test** | **p.value** |
| --- | --- | --- | --- | --- | --- | --- | --- | --- | --- |
| GREB1 | 0.081 | 1.1 (0.72-1.6) | 1.10 | 0.72 | 1.6 | | 0.15 | | 0.70 |
| CA12 | -0.010 | 0.99 (0.81-1.2) | 0.99 | 0.81 | 1.2 | | 0.01 | | 0.93 |
| SLC9A3R1 | 0.100 | 1.1 (0.7-1.8) | 1.10 | 0.70 | 1.8 | | 0.19 | | 0.66 |
| MYB | -0.260 | 0.77 (0.29-2.1) | 0.77 | 0.29 | 2.1 | | 0.26 | | 0.61 |
| ANXA9 | 0.006 | 1 (0.59-1.7) | 1.00 | 0.59 | 1.7 | | 0.00 | | 0.98 |
| IGFBP4 | 0.110 | 1.1 (0.66-1.9) | 1.10 | 0.66 | 1.9 | | 0.16 | | 0.69 |
| SYBU | -0.300 | 0.74 (0.49-1.1) | 0.74 | 0.49 | 1.1 | | 2.00 | | 0.15 |
| NPY1R | -0.049 | 0.95 (0.66-1.4) | 0.95 | 0.66 | 1.4 | | 0.07 | | 0.80 |
| PDZK1 | 0.300 | 1.3 (0.5-3.6) | 1.30 | 0.50 | 3.6 | | 0.34 | | 0.56 |
| NRIP1 | 0.001 | 1 (0.74-1.4) | 1.00 | 0.74 | 1.4 | | 0.00 | | 0.99 |
| MLPH | 0.098 | 1.1 (0.79-1.5) | 1.10 | 0.79 | 1.5 | | 0.33 | | 0.57 |
| HSPB8 | -0.160 | 0.85 (0.65-1.1) | 0.85 | 0.65 | 1.1 | | 1.20 | | 0.26 |
| EGR3 | -0.210 | 0.81 (0.61-1.1) | 0.81 | 0.61 | 1.1 | | 2.20 | | 0.13 |
| KRT19 | 0.095 | 1.1 (0.89-1.4) | 1.10 | 0.89 | 1.4 | | 0.78 | | 0.38 |
| LRIG1 | -0.130 | 0.88 (0.53-1.5) | 0.88 | 0.53 | 1.5 | | 0.25 | | 0.62 |
| KDM4B | -0.068 | 0.93 (0.49-1.8) | 0.93 | 0.49 | 1.8 | | 0.04 | | 0.84 |
| PGR | 0.012 | 1 (0.58-1.8) | 1.00 | 0.58 | 1.8 | | 0.00 | | 0.97 |
| RHOBTB3 | -0.009 | 0.99 (0.61-1.6) | 0.99 | 0.61 | 1.6 | | 0.00 | | 0.97 |
| TPD52L1 | -0.190 | 0.82 (0.57-1.2) | 0.82 | 0.57 | 1.2 | | 1.00 | | 0.31 |
| ELOVL2 | -0.280 | 0.75 (0.45-1.3) | 0.75 | 0.45 | 1.3 | | 1.10 | | 0.29 |
| RET | -0.210 | 0.81 (0.55-1.2) | 0.81 | 0.55 | 1.2 | | 1.10 | | 0.29 |
| TPBG | -0.058 | 0.94 (0.57-1.6) | 0.94 | 0.57 | 1.6 | | 0.05 | | 0.82 |
| TFF1 | 0.330 | 1.4 (1-1.9) | 1.40 | 1.00 | 1.9 | | 4.20 | | 0.04 |
| MAPT | 0.190 | 1.2 (0.68-2.1) | 1.20 | 0.68 | 2.1 | | 0.40 | | 0.53 |
| SCNN1A | -0.032 | 0.97 (0.77-1.2) | 0.97 | 0.77 | 1.2 | | 0.08 | | 0.78 |
| ABAT | 0.055 | 1.1 (0.71-1.6) | 1.10 | 0.71 | 1.6 | | 0.07 | | 0.79 |
| FLNB | 0.057 | 1.1 (0.54-2.1) | 1.10 | 0.54 | 2.1 | | 0.03 | | 0.87 |
| CELSR2 | -0.088 | 0.92 (0.58-1.4) | 0.92 | 0.58 | 1.4 | | 0.14 | | 0.70 |
| RAB31 | -0.180 | 0.84 (0.53-1.3) | 0.84 | 0.53 | 1.3 | | 0.56 | | 0.45 |
| MYBL1 | 0.130 | 1.1 (0.46-2.8) | 1.10 | 0.46 | 2.8 | | 0.08 | | 0.78 |
| MREG | -0.110 | 0.9 (0.53-1.5) | 0.90 | 0.53 | 1.5 | | 0.17 | | 0.68 |
| FAM102A | -0.008 | 0.99 (0.54-1.8) | 0.99 | 0.54 | 1.8 | | 0.00 | | 0.98 |
| MSMB | -0.013 | 0.99 (0.67-1.4) | 0.99 | 0.67 | 1.4 | | 0.00 | | 0.95 |
| STC2 | -0.290 | 0.74 (0.51-1.1) | 0.74 | 0.51 | 1.1 | | 2.40 | | 0.12 |
| RETREG1 | 0.150 | 1.2 (0.63-2.2) | 1.20 | 0.63 | 2.2 | | 0.22 | | 0.64 |
| SIAH2 | -0.150 | 0.86 (0.43-1.7) | 0.86 | 0.43 | 1.7 | | 0.18 | | 0.68 |
| ZNF185 | -0.160 | 0.85 (0.55-1.3) | 0.85 | 0.55 | 1.3 | | 0.51 | | 0.47 |
| SLC19A2 | 0.150 | 1.2 (0.69-2) | 1.20 | 0.69 | 2.0 | | 0.32 | | 0.57 |
| SLC1A4 | 0.017 | 1 (0.55-1.9) | 1.00 | 0.55 | 1.9 | | 0.00 | | 0.96 |
| FHL2 | -0.310 | 0.73 (0.49-1.1) | 0.73 | 0.49 | 1.1 | | 2.30 | | 0.13 |
| BCL2 | -0.079 | 0.92 (0.65-1.3) | 0.92 | 0.65 | 1.3 | | 0.19 | | 0.67 |
| PMAIP1 | 0.068 | 1.1 (0.78-1.5) | 1.10 | 0.78 | 1.5 | | 0.17 | | 0.68 |
| AREG | -0.048 | 0.95 (0.75-1.2) | 0.95 | 0.75 | 1.2 | | 0.15 | | 0.70 |
| OVOL2 | 0.490 | 1.6 (0.75-3.5) | 1.60 | 0.75 | 3.5 | | 1.50 | | 0.22 |
| TSKU | -0.160 | 0.85 (0.56-1.3) | 0.85 | 0.56 | 1.3 | | 0.55 | | 0.46 |
| ADCY9 | -0.140 | 0.87 (0.52-1.5) | 0.87 | 0.52 | 1.5 | | 0.27 | | 0.61 |
| RASGRP1 | 0.080 | 1.1 (0.75-1.6) | 1.10 | 0.75 | 1.6 | | 0.18 | | 0.67 |
| MUC1 | 0.025 | 1 (0.84-1.2) | 1.00 | 0.84 | 1.2 | | 0.06 | | 0.80 |
| KAZN | -0.170 | 0.84 (0.48-1.5) | 0.84 | 0.48 | 1.5 | | 0.36 | | 0.55 |
| SLC27A2 | 0.130 | 1.1 (0.73-1.8) | 1.10 | 0.73 | 1.8 | | 0.32 | | 0.57 |
| FKBP4 | -0.058 | 0.94 (0.5-1.8) | 0.94 | 0.50 | 1.8 | | 0.03 | | 0.86 |
| CXCL12 | -0.210 | 0.81 (0.61-1.1) | 0.81 | 0.61 | 1.1 | | 2.00 | | 0.16 |
| TMPRSS3 | -0.069 | 0.93 (0.63-1.4) | 0.93 | 0.63 | 1.4 | | 0.11 | | 0.74 |
| RARA | 0.230 | 1.3 (0.64-2.5) | 1.30 | 0.64 | 2.5 | | 0.45 | | 0.50 |
| IL17RB | 0.064 | 1.1 (0.5-2.3) | 1.10 | 0.50 | 2.3 | | 0.03 | | 0.87 |
| CBFA2T3 | -0.170 | 0.84 (0.45-1.6) | 0.84 | 0.45 | 1.6 | | 0.29 | | 0.59 |
| TFF3 | -0.097 | 0.91 (0.79-1.1) | 0.91 | 0.79 | 1.1 | | 1.70 | | 0.19 |
| UGCG | 0.170 | 1.2 (0.63-2.2) | 1.20 | 0.63 | 2.2 | | 0.29 | | 0.59 |
| CCND1 | -0.160 | 0.85 (0.54-1.3) | 0.85 | 0.54 | 1.3 | | 0.51 | | 0.47 |
| SLC22A5 | 0.290 | 1.3 (0.59-3.1) | 1.30 | 0.59 | 3.1 | | 0.48 | | 0.49 |
| WFS1 | -0.200 | 0.82 (0.52-1.3) | 0.82 | 0.52 | 1.3 | | 0.73 | | 0.39 |
| PTGES | -0.240 | 0.79 (0.51-1.2) | 0.79 | 0.51 | 1.2 | | 1.20 | | 0.28 |
| WWC1 | 0.240 | 1.3 (0.67-2.4) | 1.30 | 0.67 | 2.4 | | 0.53 | | 0.47 |
| CCN5 | -0.170 | 0.84 (0.54-1.3) | 0.84 | 0.54 | 1.3 | | 0.62 | | 0.43 |
| MYC | -0.280 | 0.76 (0.54-1.1) | 0.76 | 0.54 | 1.1 | | 2.70 | | 0.10 |
| ITPK1 | 0.110 | 1.1 (0.57-2.2) | 1.10 | 0.57 | 2.2 | | 0.10 | | 0.75 |
| TMEM164 | -0.180 | 0.83 (0.5-1.4) | 0.83 | 0.50 | 1.4 | | 0.49 | | 0.48 |
| ARL3 | -0.370 | 0.69 (0.36-1.3) | 0.69 | 0.36 | 1.3 | | 1.30 | | 0.25 |
| MED13L | 0.030 | 1 (0.6-1.8) | 1.00 | 0.60 | 1.8 | | 0.01 | | 0.91 |
| SEMA3B | 0.130 | 1.1 (0.82-1.6) | 1.10 | 0.82 | 1.6 | | 0.63 | | 0.43 |
| KRT18 | 0.086 | 1.1 (0.57-2.1) | 1.10 | 0.57 | 2.1 | | 0.07 | | 0.79 |
| SLC16A1 | 0.110 | 1.1 (0.7-1.8) | 1.10 | 0.70 | 1.8 | | 0.21 | | 0.64 |
| TJP3 | 0.240 | 1.3 (0.52-3.1) | 1.30 | 0.52 | 3.1 | | 0.29 | | 0.59 |
| SLC26A2 | -0.055 | 0.95 (0.55-1.6) | 0.95 | 0.55 | 1.6 | | 0.04 | | 0.84 |
| FCMR | 0.031 | 1 (0.73-1.5) | 1.00 | 0.73 | 1.5 | | 0.03 | | 0.86 |
| SULT2B1 | 0.056 | 1.1 (0.76-1.5) | 1.10 | 0.76 | 1.5 | | 0.12 | | 0.73 |
| SNX24 | -0.110 | 0.9 (0.41-2) | 0.90 | 0.41 | 2.0 | | 0.07 | | 0.79 |
| TFAP2C | -0.053 | 0.95 (0.64-1.4) | 0.95 | 0.64 | 1.4 | | 0.07 | | 0.80 |
| TTC39A | 0.200 | 1.2 (0.74-2) | 1.20 | 0.74 | 2.0 | | 0.59 | | 0.44 |
| GJA1 | -0.170 | 0.84 (0.6-1.2) | 0.84 | 0.60 | 1.2 | | 0.94 | | 0.33 |
| PRSS23 | -0.180 | 0.83 (0.63-1.1) | 0.83 | 0.63 | 1.1 | | 1.70 | | 0.19 |
| OLFM1 | -0.290 | 0.75 (0.43-1.3) | 0.75 | 0.43 | 1.3 | | 1.00 | | 0.31 |
| RAPGEFL1 | 0.220 | 1.2 (0.68-2.3) | 1.20 | 0.68 | 2.3 | | 0.49 | | 0.48 |
| ASB13 | 0.029 | 1 (0.42-2.5) | 1.00 | 0.42 | 2.5 | | 0.00 | | 0.95 |
| TIPARP | -0.140 | 0.87 (0.64-1.2) | 0.87 | 0.64 | 1.2 | | 0.79 | | 0.37 |
| ABCA3 | 0.086 | 1.1 (0.6-2) | 1.10 | 0.60 | 2.0 | | 0.08 | | 0.78 |
| FRK | -0.920 | 0.4 (0.13-1.2) | 0.40 | 0.13 | 1.2 | | 2.60 | | 0.11 |
| DHRS2 | -0.018 | 0.98 (0.6-1.6) | 0.98 | 0.60 | 1.6 | | 0.01 | | 0.94 |
| AQP3 | -0.095 | 0.91 (0.63-1.3) | 0.91 | 0.63 | 1.3 | | 0.27 | | 0.61 |
| KCNK15 | -0.052 | 0.95 (0.69-1.3) | 0.95 | 0.69 | 1.3 | | 0.10 | | 0.76 |
| TGIF2 | 0.150 | 1.2 (0.59-2.3) | 1.20 | 0.59 | 2.3 | | 0.18 | | 0.67 |
| FOXC1 | -0.240 | 0.79 (0.49-1.3) | 0.79 | 0.49 | 1.3 | | 0.95 | | 0.33 |
| ELF3 | 0.037 | 1 (0.79-1.4) | 1.00 | 0.79 | 1.4 | | 0.07 | | 0.79 |
| REEP1 | -0.110 | 0.9 (0.51-1.6) | 0.90 | 0.51 | 1.6 | | 0.15 | | 0.70 |
| PEX11A | -0.250 | 0.78 (0.48-1.3) | 0.78 | 0.48 | 1.3 | | 1.10 | | 0.30 |
| PODXL | -0.270 | 0.76 (0.51-1.1) | 0.76 | 0.51 | 1.1 | | 1.80 | | 0.18 |
| KLF4 | -0.250 | 0.78 (0.57-1.1) | 0.78 | 0.57 | 1.1 | | 2.40 | | 0.12 |
| BAG1 | -0.240 | 0.79 (0.39-1.6) | 0.79 | 0.39 | 1.6 | | 0.47 | | 0.49 |
| CELSR1 | -0.062 | 0.94 (0.63-1.4) | 0.94 | 0.63 | 1.4 | | 0.10 | | 0.76 |
| PLAAT3 | -0.470 | 0.62 (0.39-1) | 0.62 | 0.39 | 1.0 | | 3.90 | | 0.05 |
| SLC7A5 | -0.130 | 0.88 (0.62-1.2) | 0.88 | 0.62 | 1.2 | | 0.51 | | 0.48 |
| MPPED2 | -0.200 | 0.82 (0.61-1.1) | 0.82 | 0.61 | 1.1 | | 1.80 | | 0.18 |
| TIAM1 | -0.110 | 0.89 (0.69-1.2) | 0.89 | 0.69 | 1.2 | | 0.73 | | 0.39 |
| CLDN7 | 0.031 | 1 (0.6-1.8) | 1.00 | 0.60 | 1.8 | | 0.01 | | 0.91 |
| MYOF | -0.170 | 0.84 (0.56-1.3) | 0.84 | 0.56 | 1.3 | | 0.66 | | 0.42 |
| RBBP8 | -0.140 | 0.87 (0.51-1.5) | 0.87 | 0.51 | 1.5 | | 0.26 | | 0.61 |
| OLFML3 | -0.210 | 0.81 (0.57-1.2) | 0.81 | 0.57 | 1.2 | | 1.30 | | 0.26 |
| GFRA1 | -0.086 | 0.92 (0.54-1.6) | 0.92 | 0.54 | 1.6 | | 0.10 | | 0.75 |
| FARP1 | 0.100 | 1.1 (0.61-2) | 1.10 | 0.61 | 2.0 | | 0.11 | | 0.74 |
| SVIL | -0.082 | 0.92 (0.59-1.4) | 0.92 | 0.59 | 1.4 | | 0.13 | | 0.72 |
| TGM2 | 0.023 | 1 (0.75-1.4) | 1.00 | 0.75 | 1.4 | | 0.02 | | 0.89 |
| DEPTOR | -0.150 | 0.86 (0.64-1.2) | 0.86 | 0.64 | 1.2 | | 0.93 | | 0.33 |
| CYP26B1 | -0.041 | 0.96 (0.68-1.4) | 0.96 | 0.68 | 1.4 | | 0.05 | | 0.82 |
| PAPSS2 | -0.160 | 0.85 (0.59-1.2) | 0.85 | 0.59 | 1.2 | | 0.77 | | 0.38 |
| SLC1A1 | -0.170 | 0.84 (0.64-1.1) | 0.84 | 0.64 | 1.1 | | 1.50 | | 0.22 |
| DLC1 | -0.075 | 0.93 (0.62-1.4) | 0.93 | 0.62 | 1.4 | | 0.14 | | 0.71 |
| JAK2 | -0.220 | 0.81 (0.42-1.5) | 0.81 | 0.42 | 1.5 | | 0.43 | | 0.51 |
| AFF1 | 0.088 | 1.1 (0.67-1.8) | 1.10 | 0.67 | 1.8 | | 0.12 | | 0.73 |
| KLK10 | 0.035 | 1 (0.89-1.2) | 1.00 | 0.89 | 1.2 | | 0.20 | | 0.66 |
| P2RY2 | 0.110 | 1.1 (0.68-1.8) | 1.10 | 0.68 | 1.8 | | 0.18 | | 0.67 |
| BLVRB | 0.160 | 1.2 (0.6-2.3) | 1.20 | 0.60 | 2.3 | | 0.22 | | 0.64 |
| CISH | -0.089 | 0.91 (0.59-1.4) | 0.91 | 0.59 | 1.4 | | 0.15 | | 0.70 |
| GLA | -0.620 | 0.54 (0.26-1.1) | 0.54 | 0.26 | 1.1 | | 2.70 | | 0.10 |
| ADD3 | -0.190 | 0.83 (0.53-1.3) | 0.83 | 0.53 | 1.3 | | 0.71 | | 0.40 |
| PDLIM3 | 0.049 | 1.1 (0.74-1.5) | 1.10 | 0.74 | 1.5 | | 0.07 | | 0.78 |
| MINDY1 | 0.003 | 1 (0.58-1.7) | 1.00 | 0.58 | 1.7 | | 0.00 | | 0.99 |
| FOS | -0.190 | 0.83 (0.67-1) | 0.83 | 0.67 | 1.0 | | 3.10 | | 0.08 |
| KRT8 | 0.038 | 1 (0.58-1.9) | 1.00 | 0.58 | 1.9 | | 0.02 | | 0.90 |
| SLC37A1 | -0.120 | 0.89 (0.39-2) | 0.89 | 0.39 | 2.0 | | 0.08 | | 0.77 |
| B4GALT1 | -0.017 | 0.98 (0.54-1.8) | 0.98 | 0.54 | 1.8 | | 0.00 | | 0.96 |
| CALCR | 0.180 | 1.2 (0.11-13) | 1.20 | 0.11 | 13.0 | | 0.02 | | 0.88 |
| ESRP2 | -0.033 | 0.97 (0.54-1.7) | 0.97 | 0.54 | 1.7 | | 0.01 | | 0.91 |
| IGF1R | 0.034 | 1 (0.66-1.6) | 1.00 | 0.66 | 1.6 | | 0.02 | | 0.88 |
| NBL1 | -0.210 | 0.81 (0.61-1.1) | 0.81 | 0.61 | 1.1 | | 1.90 | | 0.17 |
| SFN | -0.011 | 0.99 (0.84-1.2) | 0.99 | 0.84 | 1.2 | | 0.02 | | 0.90 |
| OPN3 | -0.072 | 0.93 (0.57-1.5) | 0.93 | 0.57 | 1.5 | | 0.08 | | 0.77 |
| ABHD2 | -0.180 | 0.84 (0.57-1.2) | 0.84 | 0.57 | 1.2 | | 0.79 | | 0.37 |
| AR | -0.055 | 0.95 (0.62-1.4) | 0.95 | 0.62 | 1.4 | | 0.06 | | 0.80 |
| SLC39A6 | 0.050 | 1.1 (0.66-1.7) | 1.10 | 0.66 | 1.7 | | 0.04 | | 0.83 |
| SYT12 | -0.057 | 0.94 (0.8-1.1) | 0.94 | 0.80 | 1.1 | | 0.49 | | 0.48 |
| CD44 | 0.170 | 1.2 (0.74-1.9) | 1.20 | 0.74 | 1.9 | | 0.53 | | 0.47 |
| MED24 | 0.620 | 1.9 (0.59-5.8) | 1.90 | 0.59 | 5.8 | | 1.10 | | 0.29 |
| BCL11B | 0.140 | 1.2 (0.69-1.9) | 1.20 | 0.69 | 1.9 | | 0.31 | | 0.58 |
| CANT1 | 0.049 | 1.1 (0.57-1.9) | 1.10 | 0.57 | 1.9 | | 0.02 | | 0.87 |
| KRT13 | -0.009 | 0.99 (0.67-1.5) | 0.99 | 0.67 | 1.5 | | 0.00 | | 0.96 |
| KRT15 | 0.097 | 1.1 (0.78-1.6) | 1.10 | 0.78 | 1.6 | | 0.31 | | 0.58 |
| TOB1 | -0.430 | 0.65 (0.43-0.99) | 0.65 | 0.43 | 1.0 | | 4.10 | | 0.04 |
| SLC7A2 | 0.065 | 1.1 (0.79-1.4) | 1.10 | 0.79 | 1.4 | | 0.17 | | 0.68 |
| LAD1 | 0.008 | 1 (0.71-1.4) | 1.00 | 0.71 | 1.4 | | 0.00 | | 0.97 |
| TUBB2B | 0.190 | 1.2 (0.73-2) | 1.20 | 0.73 | 2.0 | | 0.54 | | 0.46 |
| TBC1D30 | -0.330 | 0.72 (0.28-1.9) | 0.72 | 0.28 | 1.9 | | 0.46 | | 0.50 |
| SEC14L2 | 0.018 | 1 (0.67-1.5) | 1.00 | 0.67 | 1.5 | | 0.01 | | 0.93 |
| ENDOD1 | -0.160 | 0.85 (0.6-1.2) | 0.85 | 0.60 | 1.2 | | 0.82 | | 0.37 |
| HR | -0.150 | 0.86 (0.54-1.4) | 0.86 | 0.54 | 1.4 | | 0.41 | | 0.52 |
| SCARB1 | -0.260 | 0.77 (0.46-1.3) | 0.77 | 0.46 | 1.3 | | 1.00 | | 0.32 |
| NCOR2 | 0.006 | 1 (0.51-2) | 1.00 | 0.51 | 2.0 | | 0.00 | | 0.99 |
| RHOD | -0.420 | 0.65 (0.36-1.2) | 0.65 | 0.36 | 1.2 | | 2.00 | | 0.16 |
| INPP5F | -0.130 | 0.88 (0.53-1.5) | 0.88 | 0.53 | 1.5 | | 0.23 | | 0.63 |
| PPIF | -0.230 | 0.79 (0.46-1.4) | 0.79 | 0.46 | 1.4 | | 0.71 | | 0.40 |
| DHRS3 | 0.064 | 1.1 (0.76-1.5) | 1.10 | 0.76 | 1.5 | | 0.13 | | 0.72 |
| FDFT1 | -0.100 | 0.9 (0.47-1.7) | 0.90 | 0.47 | 1.7 | | 0.09 | | 0.76 |
| GAB2 | -0.041 | 0.96 (0.58-1.6) | 0.96 | 0.58 | 1.6 | | 0.03 | | 0.87 |
| UNC119 | 0.140 | 1.2 (0.47-2.8) | 1.20 | 0.47 | 2.8 | | 0.10 | | 0.75 |
| KLF10 | -0.130 | 0.88 (0.57-1.4) | 0.88 | 0.57 | 1.4 | | 0.35 | | 0.55 |
| HES1 | -0.100 | 0.9 (0.57-1.4) | 0.90 | 0.57 | 1.4 | | 0.20 | | 0.66 |
| FKBP5 | 0.013 | 1 (0.72-1.4) | 1.00 | 0.72 | 1.4 | | 0.01 | | 0.94 |
| SLC2A1 | -0.120 | 0.88 (0.54-1.4) | 0.88 | 0.54 | 1.4 | | 0.24 | | 0.62 |
| AMFR | 0.059 | 1.1 (0.52-2.1) | 1.10 | 0.52 | 2.1 | | 0.03 | | 0.87 |
| NADSYN1 | 0.150 | 1.2 (0.45-2.9) | 1.20 | 0.45 | 2.9 | | 0.09 | | 0.76 |
| INHBB | -0.029 | 0.97 (0.75-1.3) | 0.97 | 0.75 | 1.3 | | 0.05 | | 0.83 |
| BHLHE40 | -0.130 | 0.88 (0.64-1.2) | 0.88 | 0.64 | 1.2 | | 0.66 | | 0.42 |
| CALB2 | -0.250 | 0.78 (0.47-1.3) | 0.78 | 0.47 | 1.3 | | 0.98 | | 0.32 |
| FASN | 0.160 | 1.2 (0.67-2) | 1.20 | 0.67 | 2.0 | | 0.32 | | 0.57 |
| CHPT1 | -0.150 | 0.86 (0.57-1.3) | 0.86 | 0.57 | 1.3 | | 0.53 | | 0.47 |
| MYBBP1A | 0.750 | 2.1 (0.75-6) | 2.10 | 0.75 | 6.0 | | 2.00 | | 0.16 |
| ELOVL5 | 0.240 | 1.3 (0.67-2.4) | 1.30 | 0.67 | 2.4 | | 0.54 | | 0.46 |
| DYNLT3 | -0.200 | 0.82 (0.45-1.5) | 0.82 | 0.45 | 1.5 | | 0.43 | | 0.51 |
| ABLIM1 | -0.062 | 0.94 (0.61-1.5) | 0.94 | 0.61 | 1.5 | | 0.08 | | 0.78 |
| SOX3 | 0.220 | 1.2 (0.94-1.6) | 1.20 | 0.94 | 1.6 | | 2.40 | | 0.12 |
| SLC24A3 | 0.053 | 1.1 (0.75-1.5) | 1.10 | 0.75 | 1.5 | | 0.09 | | 0.76 |
| RAB17 | 0.015 | 1 (0.57-1.8) | 1.00 | 0.57 | 1.8 | | 0.00 | | 0.96 |
| MAST4 | -0.210 | 0.81 (0.41-1.6) | 0.81 | 0.41 | 1.6 | | 0.39 | | 0.53 |
| KCNK5 | -0.099 | 0.91 (0.57-1.4) | 0.91 | 0.57 | 1.4 | | 0.18 | | 0.67 |
| ELF1 | -0.110 | 0.9 (0.6-1.3) | 0.90 | 0.60 | 1.3 | | 0.27 | | 0.61 |
| RPS6KA2 | -0.280 | 0.76 (0.47-1.2) | 0.76 | 0.47 | 1.2 | | 1.30 | | 0.25 |
| ISG20L2 | -0.140 | 0.87 (0.42-1.8) | 0.87 | 0.42 | 1.8 | | 0.14 | | 0.71 |
| IL6ST | 0.076 | 1.1 (0.61-1.9) | 1.10 | 0.61 | 1.9 | | 0.07 | | 0.79 |
| SYNGR1 | -0.250 | 0.78 (0.51-1.2) | 0.78 | 0.51 | 1.2 | | 1.30 | | 0.26 |
| SH3BP5 | -0.240 | 0.78 (0.53-1.2) | 0.78 | 0.53 | 1.2 | | 1.50 | | 0.22 |
| ALDH3B1 | 0.034 | 1 (0.74-1.4) | 1.00 | 0.74 | 1.4 | | 0.04 | | 0.84 |
| THSD4 | -0.090 | 0.91 (0.59-1.4) | 0.91 | 0.59 | 1.4 | | 0.17 | | 0.68 |
| CLIC3 | -0.150 | 0.86 (0.62-1.2) | 0.86 | 0.62 | 1.2 | | 0.89 | | 0.35 |
| NXT1 | 0.005 | 1 (0.44-2.3) | 1.00 | 0.44 | 2.3 | | 0.00 | | 0.99 |
| NAV2 | -0.077 | 0.93 (0.63-1.4) | 0.93 | 0.63 | 1.4 | | 0.15 | | 0.70 |
| RRP12 | 0.410 | 1.5 (0.64-3.6) | 1.50 | 0.64 | 3.6 | | 0.89 | | 0.35 |
| ADCY1 | 0.011 | 1 (0.65-1.6) | 1.00 | 0.65 | 1.6 | | 0.00 | | 0.96 |
| DHCR7 | -0.440 | 0.64 (0.38-1.1) | 0.64 | 0.38 | 1.1 | | 2.60 | | 0.11 |
| MICB | -0.160 | 0.85 (0.49-1.5) | 0.85 | 0.49 | 1.5 | | 0.32 | | 0.57 |
| AKAP1 | -0.150 | 0.86 (0.52-1.4) | 0.86 | 0.52 | 1.4 | | 0.32 | | 0.57 |
| ASS1 | -0.260 | 0.77 (0.47-1.3) | 0.77 | 0.47 | 1.3 | | 1.10 | | 0.30 |
| GPER1 | -0.270 | 0.76 (0.52-1.1) | 0.76 | 0.52 | 1.1 | | 2.10 | | 0.15 |
| LLGL2 | 0.560 | 1.8 (0.68-4.5) | 1.80 | 0.68 | 4.5 | | 1.40 | | 0.25 |
| AGR2 | 0.010 | 1 (0.83-1.2) | 1.00 | 0.83 | 1.2 | | 0.01 | | 0.92 |
| PDCD4 | -0.110 | 0.9 (0.54-1.5) | 0.90 | 0.54 | 1.5 | | 0.18 | | 0.67 |
| DNAJC12 | -0.830 | 0.44 (0.12-1.6) | 0.44 | 0.12 | 1.6 | | 1.60 | | 0.21 |
| TSPAN13 | -0.480 | 0.62 (0.41-0.94) | 0.62 | 0.41 | 0.9 | | 5.10 | | 0.02 |
| PKP3 | -0.310 | 0.73 (0.47-1.2) | 0.73 | 0.47 | 1.2 | | 1.80 | | 0.18 |
| ISG20 | -0.092 | 0.91 (0.55-1.5) | 0.91 | 0.55 | 1.5 | | 0.12 | | 0.72 |
| SERPINA3 | -2.300 | 0.1 (1e-04-97) | 0.10 | 0.00 | 97.0 | | 0.43 | | 0.51 |
| GJB3 | -0.069 | 0.93 (0.76-1.1) | 0.93 | 0.76 | 1.1 | | 0.43 | | 0.51 |
| PRLR | 0.330 | 1.4 (0.75-2.6) | 1.40 | 0.75 | 2.6 | | 1.10 | | 0.29 |
| SERPINA5 | -0.870 | 0.42 (0.12-1.5) | 0.42 | 0.12 | 1.5 | | 1.80 | | 0.18 |
| IMPA2 | -0.040 | 0.96 (0.63-1.5) | 0.96 | 0.63 | 1.5 | | 0.04 | | 0.85 |
| CDH1 | -0.062 | 0.94 (0.58-1.5) | 0.94 | 0.58 | 1.5 | | 0.06 | | 0.80 |
| EMP2 | -0.052 | 0.95 (0.61-1.5) | 0.95 | 0.61 | 1.5 | | 0.05 | | 0.82 |
| DLG5 | -0.019 | 0.98 (0.65-1.5) | 0.98 | 0.65 | 1.5 | | 0.01 | | 0.93 |
| PCP4 | -0.066 | 0.94 (0.72-1.2) | 0.94 | 0.72 | 1.2 | | 0.23 | | 0.63 |
| NAB2 | -0.024 | 0.98 (0.68-1.4) | 0.98 | 0.68 | 1.4 | | 0.02 | | 0.89 |
| LSR | 0.240 | 1.3 (0.68-2.4) | 1.30 | 0.68 | 2.4 | | 0.55 | | 0.46 |
| CACNA2D2 | -0.049 | 0.95 (0.68-1.3) | 0.95 | 0.68 | 1.3 | | 0.08 | | 0.78 |
| CA2 | -0.100 | 0.9 (0.65-1.2) | 0.90 | 0.65 | 1.2 | | 0.41 | | 0.52 |
| ASCL1 | -1.600 | 0.19 (0.0054-6.9) | 0.19 | 0.01 | 6.9 | | 0.81 | | 0.37 |
| ACOX2 | -0.028 | 0.97 (0.52-1.8) | 0.97 | 0.52 | 1.8 | | 0.01 | | 0.93 |
| PERP | 0.041 | 1 (0.72-1.5) | 1.00 | 0.72 | 1.5 | | 0.05 | | 0.83 |
| UGDH | -0.250 | 0.78 (0.47-1.3) | 0.78 | 0.47 | 1.3 | | 0.93 | | 0.34 |
| KLK11 | 0.062 | 1.1 (0.92-1.2) | 1.10 | 0.92 | 1.2 | | 0.66 | | 0.42 |
| PLAC1 | 0.230 | 1.3 (0.24-6.6) | 1.30 | 0.24 | 6.6 | | 0.07 | | 0.79 |
| DCXR | -0.064 | 0.94 (0.57-1.5) | 0.94 | 0.57 | 1.5 | | 0.07 | | 0.80 |
| CCNA1 | -0.044 | 0.96 (0.68-1.4) | 0.96 | 0.68 | 1.4 | | 0.06 | | 0.80 |
| STIL | 0.920 | 2.5 (0.81-7.7) | 2.50 | 0.81 | 7.7 | | 2.60 | | 0.11 |
| ZFP36 | -0.250 | 0.78 (0.58-1.1) | 0.78 | 0.58 | 1.1 | | 2.70 | | 0.10 |
| CAV1 | -0.150 | 0.86 (0.65-1.1) | 0.86 | 0.65 | 1.1 | | 1.20 | | 0.28 |
| HOMER2 | 0.090 | 1.1 (0.57-2.1) | 1.10 | 0.57 | 2.1 | | 0.07 | | 0.79 |
| BTG3 | 0.130 | 1.1 (0.57-2.3) | 1.10 | 0.57 | 2.3 | | 0.14 | | 0.71 |
| GAL | -0.700 | 0.49 (0.096-2.5) | 0.49 | 0.10 | 2.5 | | 0.71 | | 0.40 |
| ETFB | -0.039 | 0.96 (0.52-1.8) | 0.96 | 0.52 | 1.8 | | 0.02 | | 0.90 |
| IGSF1 | -0.027 | 0.97 (0.84-1.1) | 0.97 | 0.84 | 1.1 | | 0.12 | | 0.73 |
| HPRT1 | -0.300 | 0.74 (0.33-1.7) | 0.74 | 0.33 | 1.7 | | 0.52 | | 0.47 |
| CDC6 | 0.180 | 1.2 (0.63-2.3) | 1.20 | 0.63 | 2.3 | | 0.31 | | 0.58 |
| S100A9 | 0.004 | 1 (0.78-1.3) | 1.00 | 0.78 | 1.3 | | 0.00 | | 0.98 |
| SLC29A1 | 0.034 | 1 (0.58-1.9) | 1.00 | 0.58 | 1.9 | | 0.01 | | 0.91 |
| KIF20A | 0.530 | 1.7 (1.1-2.6) | 1.70 | 1.10 | 2.6 | | 5.60 | | 0.02 |
| DNAJC1 | -0.240 | 0.79 (0.45-1.4) | 0.79 | 0.45 | 1.4 | | 0.74 | | 0.39 |
| TPSAB1 | -0.270 | 0.76 (0.53-1.1) | 0.76 | 0.53 | 1.1 | | 2.20 | | 0.14 |
| TSTA3 | 0.280 | 1.3 (0.59-3) | 1.30 | 0.59 | 3.0 | | 0.45 | | 0.50 |
| FGFR3 | -0.004 | 1 (0.73-1.4) | 1.00 | 0.73 | 1.4 | | 0.00 | | 0.98 |
| SGK1 | -0.059 | 0.94 (0.66-1.4) | 0.94 | 0.66 | 1.4 | | 0.10 | | 0.75 |
| ID2 | -0.210 | 0.81 (0.51-1.3) | 0.81 | 0.51 | 1.3 | | 0.71 | | 0.40 |
| GALE | 0.120 | 1.1 (0.79-1.6) | 1.10 | 0.79 | 1.6 | | 0.45 | | 0.50 |
| BATF | 0.012 | 1 (0.75-1.4) | 1.00 | 0.75 | 1.4 | | 0.01 | | 0.94 |
| MAPK13 | 0.310 | 1.4 (0.74-2.5) | 1.40 | 0.74 | 2.5 | | 0.97 | | 0.33 |
| FABP5 | 0.086 | 1.1 (0.72-1.6) | 1.10 | 0.72 | 1.6 | | 0.17 | | 0.68 |
| MEST | -0.150 | 0.86 (0.44-1.7) | 0.86 | 0.44 | 1.7 | | 0.20 | | 0.66 |
| JAK1 | -0.140 | 0.87 (0.51-1.5) | 0.87 | 0.51 | 1.5 | | 0.26 | | 0.61 |
| CYP4F11 | -0.084 | 0.92 (0.71-1.2) | 0.92 | 0.71 | 1.2 | | 0.41 | | 0.52 |
| CPE | -0.270 | 0.77 (0.59-0.99) | 0.77 | 0.59 | 1.0 | | 4.20 | | 0.04 |
| XRCC3 | 0.420 | 1.5 (0.67-3.5) | 1.50 | 0.67 | 3.5 | | 1.00 | | 0.32 |
| CXCL14 | -0.100 | 0.9 (0.81-1) | 0.90 | 0.81 | 1.0 | | 3.10 | | 0.08 |
| SCUBE2 | -0.230 | 0.79 (0.47-1.3) | 0.79 | 0.47 | 1.3 | | 0.77 | | 0.38 |
| CDC20 | 0.330 | 1.4 (0.94-2.1) | 1.40 | 0.94 | 2.1 | | 2.70 | | 0.10 |
| GINS2 | 0.210 | 1.2 (0.72-2.1) | 1.20 | 0.72 | 2.1 | | 0.59 | | 0.44 |
| TRIM29 | -0.100 | 0.9 (0.7-1.2) | 0.90 | 0.70 | 1.2 | | 0.58 | | 0.45 |
| UNC13B | -0.028 | 0.97 (0.51-1.9) | 0.97 | 0.51 | 1.9 | | 0.01 | | 0.93 |
| LAMC2 | 0.075 | 1.1 (0.7-1.7) | 1.10 | 0.70 | 1.7 | | 0.12 | | 0.73 |
| LARGE1 | -0.054 | 0.95 (0.49-1.8) | 0.95 | 0.49 | 1.8 | | 0.03 | | 0.87 |
| SLC2A8 | -0.130 | 0.88 (0.44-1.8) | 0.88 | 0.44 | 1.8 | | 0.13 | | 0.71 |
| PLXNB1 | 0.270 | 1.3 (0.72-2.4) | 1.30 | 0.72 | 2.4 | | 0.78 | | 0.38 |
| PRKAR2B | 0.035 | 1 (0.56-1.9) | 1.00 | 0.56 | 1.9 | | 0.01 | | 0.91 |
| HSPA4L | -0.170 | 0.85 (0.49-1.5) | 0.85 | 0.49 | 1.5 | | 0.35 | | 0.55 |
| TFPI2 | -0.170 | 0.84 (0.54-1.3) | 0.84 | 0.54 | 1.3 | | 0.62 | | 0.43 |
| SERPINA1 | -0.031 | 0.97 (0.84-1.1) | 0.97 | 0.84 | 1.1 | | 0.17 | | 0.68 |
| TNNC1 | -0.040 | 0.96 (0.67-1.4) | 0.96 | 0.67 | 1.4 | | 0.05 | | 0.82 |
| HMGCS2 | 0.093 | 1.1 (0.79-1.5) | 1.10 | 0.79 | 1.5 | | 0.32 | | 0.57 |
| ALDH3A2 | -0.034 | 0.97 (0.56-1.7) | 0.97 | 0.56 | 1.7 | | 0.02 | | 0.90 |
| CD9 | -0.140 | 0.87 (0.59-1.3) | 0.87 | 0.59 | 1.3 | | 0.45 | | 0.50 |
| IDH2 | 0.015 | 1 (0.48-2.2) | 1.00 | 0.48 | 2.2 | | 0.00 | | 0.97 |
| SORD | -0.270 | 0.77 (0.56-1.1) | 0.77 | 0.56 | 1.1 | | 2.70 | | 0.10 |
| MDK | 0.037 | 1 (0.81-1.3) | 1.00 | 0.81 | 1.3 | | 0.09 | | 0.77 |
| PTGER3 | -0.360 | 0.7 (0.26-1.9) | 0.70 | 0.26 | 1.9 | | 0.51 | | 0.47 |
| RABEP1 | -0.036 | 0.96 (0.47-2) | 0.96 | 0.47 | 2.0 | | 0.01 | | 0.92 |
| SNX10 | -0.018 | 0.98 (0.64-1.5) | 0.98 | 0.64 | 1.5 | | 0.01 | | 0.94 |
| METTL3 | 0.084 | 1.1 (0.54-2.2) | 1.10 | 0.54 | 2.2 | | 0.06 | | 0.81 |
| PLK4 | 0.760 | 2.1 (0.81-5.7) | 2.10 | 0.81 | 5.7 | | 2.40 | | 0.13 |
| COX6C | -0.170 | 0.85 (0.42-1.7) | 0.85 | 0.42 | 1.7 | | 0.22 | | 0.64 |
| ST14 | 0.008 | 1 (0.56-1.8) | 1.00 | 0.56 | 1.8 | | 0.00 | | 0.98 |
| MOCS2 | -0.290 | 0.75 (0.34-1.7) | 0.75 | 0.34 | 1.7 | | 0.52 | | 0.47 |
| NMU | 0.190 | 1.2 (1-1.4) | 1.20 | 1.00 | 1.4 | | 5.60 | | 0.02 |
| TH | -0.600 | 0.55 (0.16-1.9) | 0.55 | 0.16 | 1.9 | | 0.89 | | 0.34 |
| RNASEH2A | 0.270 | 1.3 (0.58-3) | 1.30 | 0.58 | 3.0 | | 0.41 | | 0.52 |
| CHST8 | -1.200 | 0.3 (0.054-1.7) | 0.30 | 0.05 | 1.7 | | 1.80 | | 0.17 |
| TST | 0.200 | 1.2 (0.66-2.2) | 1.20 | 0.66 | 2.2 | | 0.40 | | 0.53 |
| TOP2A | 0.390 | 1.5 (1-2.2) | 1.50 | 1.00 | 2.2 | | 4.00 | | 0.05 |
| CKB | 0.140 | 1.1 (0.79-1.7) | 1.10 | 0.79 | 1.7 | | 0.53 | | 0.46 |
| LTF | -0.200 | 0.82 (0.6-1.1) | 0.82 | 0.60 | 1.1 | | 1.50 | | 0.22 |
| DUSP2 | -0.160 | 0.85 (0.67-1.1) | 0.85 | 0.67 | 1.1 | | 1.60 | | 0.21 |
| PTPN6 | -0.099 | 0.91 (0.42-2) | 0.91 | 0.42 | 2.0 | | 0.06 | | 0.80 |
| ATP2B4 | 0.110 | 1.1 (0.66-1.9) | 1.10 | 0.66 | 1.9 | | 0.18 | | 0.67 |
| ST6GALNAC2 | 0.220 | 1.3 (0.78-2) | 1.30 | 0.78 | 2.0 | | 0.87 | | 0.35 |
