# Supplementary material for: Identification and validation of eight estrogen-related genes for predicting prognosis of papillary thyroid cancer
Source: Aging (Albany NY). 2023 Mar 13;15(5):1668–84. doi: 10.18632/aging.204582 (PMC10042678; doi:10.18632/aging.204582)
Supplement: Supplementary Tables 3-5 [file aging-15-204582-s004.pdf]

SUPPLEMENTARY TABLES

Supplementary Table 3. Clinical data statistics for the whole set, test set and training set.

Variable	Whole	Test set	Training set
n	501	250	251
**PFI (%)**			
0	449 (89.6)	225 (90.0)	224 (89.2)
1	52 (10.4)	25 (10.0)	27 (10.8)
**Age (mean (SD))**	47.34 (15.85)	47.76 (15.73)	46.92 (15.99)
**Gender (%)**			
Female	366 (73.1)	180 (72.0)	186 (74.1)
Male	135 (26.9)	70 (28.0)	65 (25.9)
**T (%)**			
T1	142 (28.3)	77 (30.8)	65 (25.9)
T2	164 (32.7)	82 (32.8)	82 (32.7)
T3	170 (33.9)	78 (31.2)	92 (36.7)
T4	23 (4.6)	13 (5.2)	10 (4.0)
TX	2 (0.4)	0 (0.0)	2 (0.8)
**M (%)**			
Unknown	1 (0.2)	1 (0.4)	0 (0.0)
M0	282 (56.3)	148 (59.2)	134 (53.4)
M1	9 (1.8)	3 (1.2)	6 (2.4)
MX	209 (41.7)	98 (39.2)	111 (44.2)
***N* (%)**			
N0	229 (45.7)	127 (50.8)	102 (40.6)
N1	222 (44.3)	103 (41.2)	119 (47.4)
NX	50 (10.0)	20 (8.0)	30 (12.0)
**Stage (%)**			
Unknown	2 (0.4)	1 (0.4)	1 (0.4)
Stage I	281 (56.1)	139 (55.6)	142 (56.6)
Stage II	52 (10.4)	28 (11.2)	24 (9.6)
Stage III	111 (22.2)	53 (21.2)	58 (23.1)
Stage IV	55 (11.0)	29 (11.6)	26 (10.4)

Supplementary Table 4. The siRNA sequences used in this study are as follows.

siRNA name	siRNA sequence	
	Sense (5′-3′)	Antisense (5′-3′)
**siNMU#1**	5′-GAACAAGAACU ACAAUUGUUU-3′	5′-ACAAUUGUAGUU CUUGUUCUG-3′
**siNMU#2**	5′-GCCGUAUAGUGA UGGAGAUUU-3′	5′-AUCUCCAUCACU AUACGGCAA-3′

The sequence of Negative control (NC) group of siRNA was provided by Tsingke.

Supplementary Table 5. Primer sequences used for RT-qPCR in this study.

Gene name	Primer sequence	
	Forward Primer	Reverse Primer
**β-actin**	CCCAGCCATGTACGTTGCTA	TCACCGGAGTCCATCACGAT
**NMU**	CTCAGGCATCCAACGCACT	GACTTGCCCAACTTCTGTGTC
